# Hydroxyurea induces an oxidative stress response that triggers ER expansion and cytoplasmic protein aggregation

**DOI:** 10.1371/journal.pbio.3003493

**Published:** 2025-11-19

**Authors:** Ana Sánchez-Molina, Manuel Bernal, Joel D. Posligua-García, Antonio J. Pérez-Pulido, Laura de Cubas, Elena Hidalgo, M.-Henar Valdivieso, Silvia Salas-Pino, Rafael R. Daga

**Affiliations:** 1 Centro Andaluz de Biología del Desarrollo, Departamento de Biología Molecular e Ingeniería Bioquímica, Universidad Pablo de Olavide, Seville, Spain; 2 Departamento de Biología Molecular y Bioquímica, Facultad de Ciencias, Universidad de Málaga, Málaga, Spain; 3 Instituto de Investigación Biomédica de Málaga y Plataforma en Nanomedicina, IBIMA Plataforma BIONAND, Málaga, Spain; 4 CIBER de Enfermedades Raras (CIBERER), Madrid, Spain; 5 Oxidative Stress and Cell Cycle Group, Universitat Pompeu Fabra, Barcelona, Spain; 6 Departamento de Microbiología y Genética, Universidad de Salamanca, Salamanca, Spain; 7 Instituto de Biología Funcional y Genómica, Consejo Superior de Investigaciones Científicas, Salamanca, Spain; University of Kaiserslautern: Technische Universitat Kaiserslautern, GERMANY

## Abstract

The endoplasmic reticulum (ER) lumen provides the proper redox environment for disulfide bond formation, which is essential for the correct folding of proteins entering the secretory pathway and forming membranes. However, the precise mechanisms by which disruptions in protein folding within the ER activate proteostatic mechanisms remain to be fully elucidated. In this study, we demonstrate that in *Schizosaccharomyces pombe* the antineoplastic agent hydroxyurea (HU) induces a transient perinuclear ER expansion, Bip1 accumulation, and the clustering of nuclear pore complexes in a specific region of the nuclear envelope. This striking phenotype is mimicked by diamide (DIA), a specific inducer of thiol stress, and can be prevented or rapidly reversed by dithiothreitol, a reducing agent, suggesting that ER expansion results from disulfide stress. Furthermore, HU or DIA treatments resulted in the accumulation of misfolded proteins in cytoplasmic foci containing Hsp104 disaggregase and Hsp70/Ssa1 chaperones. Our data show that HU impacts redox-dependent protein folding, impairs the secretory pathway, and activates specific proteostatic mechanisms in both the ER and the cytoplasm.

## Abstract 2

The ER lumen provides the proper redox environment for disulfide bond formation, which is essential for the correct folding of proteins, but the responses activated by incorrect folding remain unclear. This study shows that the cancer drug hydroxyurea disrupts redox-dependent protein folding in the ER, triggering a stress response that leads to perinuclear ER expansion and cytoplasmic protein aggregation.

## Introduction

The endoplasmic reticulum (ER) is the largest endomembrane organelle in the cell, and it is responsible for the synthesis, folding, and assembly of secretory and membrane proteins, which represent almost one third of the eukaryotic proteome [1,2]. To achieve their functional conformation, nascent proteins undergo sequential folding in the ER lumen, coordinated with post-translational modifications such as glycosylation and disulfide bond formation [3,4]. Defects in redox folding can lead to protein misfolding and aggregation, causing both ER and cellular stress [5]. To prevent this, cells monitor these processes through the coordinated action of protein disulfide isomerases (PDIs) and oxidoreductases [6,7]. When proteins fail to fold correctly in the ER, cells activate the unfolded protein response (UPR), a coordinated network of signaling cascades that collaboratively reduce overall mRNA translation, increase the folding capacity of the ER and contribute to the removal of unfolded proteins through ER-associated degradation (ERAD) and autophagy [8,9]. Additionally, in response to ER stress, the UPR activates lipid biosynthesis, prompting membrane expansion and subsequently increasing ER size, which helps mitigate folding stress [10].

Glutathione (GSH) plays a central role in redox regulation, as it counters the oxidation potential by maintaining a significant fraction of PDIs and oxidoreductases in the reduced state, and directly contributes to the reduction of disulfides along with the thioredoxin and glutaredoxin systems [11–15]. GSH also plays an important role in the biosynthesis and maturation of Iron-Sulfur (Fe-S) clusters in proteins [13,16]. In the ER, the GSH/oxidized glutathione (GSSG) ratio is around 1.5:1 to 3.3:1 [17] to facilitate disulfide bond formation and proper oxidative protein folding. In contrast, in the cytosol, disulfide bonds rarely form due to the high GSH/GSSG ratio (~100:1) [11,18,19], which favors proteins to remain in their reduced state [20]. Imbalances in the cytosolic GSH/GSSG ratio can alter global GSH/GSSG levels [21], which in turn may lead to impairments in organelle-specific redox regulation [16,22].

Hydroxyurea (HU) is a potent and reversible inhibitor of the enzyme ribonucleotide reductase (RNR) that is used as an FDA-approved antiproliferative drug in cancer therapy. In addition, HU is the primary treatment for sickle cell anemia, and it is also used as an antiviral and to treat several infectious diseases [23]. In spite of its clinical efficacy as RNR inhibitor, it is known that HU generates reactive oxygen species (ROS), interferes with iron-cluster containing enzymes, targets cells deficient in matrix metallopeptidase 2 (MMP2) [24] and might have yet other unknown off-target effects.

In this work, we show that HU triggers a transient expansion of the perinuclear ER lumen and the clustering of nuclear pore complexes (NPCs) in one region of the nuclear envelope (NE). This phenotype is mimicked by diamide (DIA), a specific thiol stress inducer. ER expansion accumulates ER-specific Hsp70 chaperone Bip1, while several other heat shock proteins (HSPs) accumulate in cytoplasmic aggregation foci, suggesting that both HU and DIA treatments impair protein folding in the ER and in the cytoplasm. During the revision of this work, a paper was published that demonstrates that HU inhibits the ERAD-luminal pathway in budding yeast by inducing ectopic disulfide bond formation in ER lumen proteins [25], which supports our observations and provides additional evidence that HU modulates cellular redox homeostasis.

## Results

### HU causes reversible NPC clustering and ER expansion independent of RNR activity inhibition

**Fig 1:**
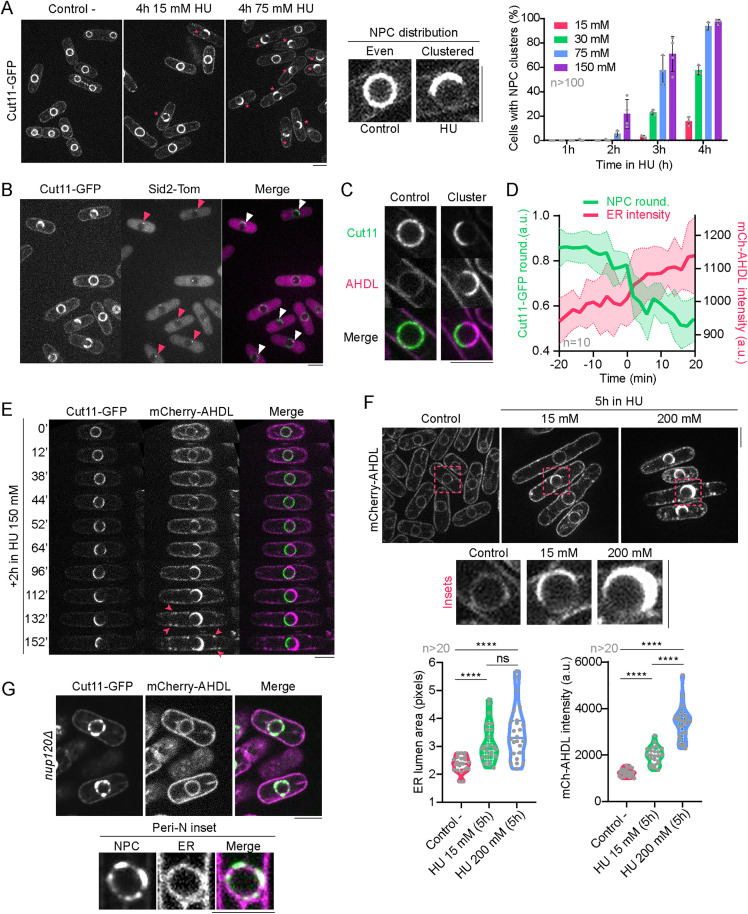
Hydroxyurea induces NPC clustering and ER expansion. **(A) Left:** Confocal microscopy images of wild-type cells expressing GFP-tagged nucleoporin Cut11 in untreated conditions (left) and after 4 hours in 15 mM HU (center) or 75 mM HU (right). Asterisks marks cells in which NPC clustering is observed. **Center:** Insets showing the even distribution of NPCs observed in an untreated cell vs. the phenotype of clustered NPCs observed upon HU treatment. **Right:** Quantification of the percentage of cells showing clustered NPCs after treatment with different concentrations of HU for the indicated times. The graph shows the mean ± SD of three independent experiments, and in each repetition n > 100 cells were accounted for each HU concentration, temperature and time point. **(B)** Images of wild-type cells expressing GFP-tagged nucleoporin Cut11 and the SPB marker Sid2-Tomato and treated with 15mM HU for 4 hours. Arrows indicate cells in which the SPB colocalizes with NPCs cluster. **(C)** Nuclear insets of confocal microscopy images showing a control nucleus in which Cut11-GFP and the luminal ER marker mCherry-AHDL colocalize along the NE, and a nucleus where Cut11-GFP and mCherry-AHDL occupy opposite sides of the nuclear periphery after a 4-hour 15 mM HU treatment. **(D)** Quantification of the loss of roundness of NPCs along the NE (from 1, full roundness, to 0.4, less than half of the circumference of the NE decorated with NPCs), which correlates with an increment in mCherry-AHDL fluorescence intensity (measured in arbitrary units) along the nuclear periphery. Graph shows the mean ± SD of 10 representative cells which develop the phenotype along the course of the experiment, during a 4-hour long incubation in 75 mM HU. The time points indicated below are normalized to the moment when N-Caps completely form (‘0′). **(E)** Timelapse depicting NPC clustering and ER expansion in a cell expressing Cut11-GFP and mCherry-AHDL after exposure to 150 mM HU for two hours before the beginning of the experiment. Arrows show the appearance of cortical ER expansions. **(F) Up:** Images showing mCherry-AHDL to compare luminal ER size and intensity in an untreated condition and after exposure to 15 mM or 200 mM HU for 5 hours. Insets show a close-up of a significant nucleus in each condition. **Below:** Quantifications of ER area, measured in pixels, and mCherry-AHDL fluorescent intensity in the nuclear periphery after a 5-hour incubation in either 15 mM or 200 mM HU, compared to the untreated condition. Graphs show violin plots with their respective mean ± SD, where at least 10 cells were accounted for in each condition. **(G)** Images and nuclear insets of the mutant strain *nup120Δ*, where NPCs tagged with Cut11-GFP are not evenly distributed along the NE while the luminal ER marker mCherry-AHDL remains unaltered in the nuclear periphery. All confocal microscopy images are SUM projections of three central Z slices. Scale bars represent 5 microns. For additional information, consult S1 Fig. Source data for this Figure and for S1 Fig can be found in S1 Data.

In research laboratories, HU is commonly used to synchronize cells in S phase. By doing so, we unexpectedly found that HU treatment in *Schizosaccharomyces pombe* resulted in an altered localization of NPCs. Incubation of cells expressing nucleoporin Cut11-GFP (a component of the NPC membrane ring) with 15 mM HU for four hours, a concentration and time known to arrest cell cycle in S phase [26,27], disrupted the even localization of NPCs and resulted in their accumulation in a localized region of the NE close to the spindle pole body (SPB, centrosome equivalent) marked with Sid2-Tom (Fig 1A, 1B; for results, see S1 Data). This previously undescribed phenotype was dose-dependent, as increasing the concentration of HU resulted in up to 97.33 ± 2.08% (n > 100 cells) of cells showing NPC clustering (Fig 1A, graph).

During HU treatment, clustered NPCs remain functional for nucleocytoplasmic transport. This was demonstrated by measuring the rate of importin-α (Imp1) transport across evenly distributed NPCs and clustered NPCs in HU-exposed cells by fluorescence recovery after photobleaching (FRAP), bleaching either the cytoplasmic or nuclear pools of Imp1-GFP, and analyzing the fluorescence recovery in the adjacent compartment (S1A Fig; for results, see S1 Data). We also determined that the translocation rate across NPCs of the transcription factor Pap1 upon hydrogen peroxide (H_2_O_2_) addition, which induces its translocation to the nucleus, and after H_2_O_2_ washout, which induces Pap1 transport back to the cytoplasm [28–30], was similar in cells with normal NPCs and cells with clustered NPCs in the presence of HU (S1B Fig).

As the primary target of HU is RNR, we checked whether NPC clustering was related to its inhibition or to the cell cycle arrest in S phase. RNR is a highly conserved tetrameric enzyme composed of two large subunits (Cdc22^R1^) and two small subunits (Suc22^R2^). Whereas Cdc22^R1^, which bears the catalytic activity, is mainly cytoplasmic, Suc22^R2^ is actively imported into the nucleus in a Spd1-dependent manner. Upon HU treatment, Spd1 is degraded, allowing cytoplasmic accumulation of Suc22^R2^ [31,32]. The appearance of clustered NPCs was unrelated to the differential nucleocytoplasmic compartmentalization of the two RNR subunits, as deletion of *spd1*, which results in both subunits constitutively localizing in the cytoplasm [32], did not alter the frequency of NPC clustering (S1C Fig). Inactivation of Cdc22^R1^ by using a thermosensitive *cdc22-M45* mutant strain and keeping it at restrictive temperature for over four hours [33,34] did not result in NPC clustering (S1D Fig). Furthermore, HU-dependent NPC clustering could be induced in G2/M blocked cells (by inactivation of *cdc25-22*, a thermosensitive allele of the mitotic activator Cdc25) (S1E Fig). Together, these results indicate that HU-induced NPC clustering is a phenomenon not related to S phase arrest nor to deoxyribonucleotide synthesis inhibition, which is the main known cellular effect of HU, and instead is the result of an undescribed off-target of this drug.

HU-induced NPC clustering phenotype was accompanied by a severe expansion of the perinuclear ER lumen in the area devoid of NPCs (Fig 1C). Timelapse confocal microscopy images showed that NPC clustering and perinuclear ER lumen enlargement occurred concomitantly (Fig 1D, 1E; for results, see S1 Data). After the initial expansion of the perinuclear ER lumen, events of cortical ER expansion were also observed when HU exposure time increased (Fig 1E, arrows). Similar to NPC clustering, HU-induced perinuclear ER enlargement was time- and dose-dependent (Fig 1F). Thus, HU induces an overall change in nuclear architecture, including expansion of perinuclear luminal ER and NPC clustering, a phenotype that we named ‘Nuclear Cap’ (N-Cap).

We next asked whether the enlarged perinuclear ER resulted from the clustering of NPCs or if, on the contrary, remodeling of the perinuclear ER was causing NPCs to cluster. For that, we used a strain deleted for nucleoporin Nup120, a component of Y-complex of the inner and outer NPC rings and whose absence results in defective and aberrantly clustered NPCs [35]. Clustered NPCs in *nup120Δ* cells showed an apparently normal perinuclear ER morphology (Fig 1G), indicating that NPC clustering per se does not cause detectable perinuclear ER asymmetry. Therefore, we concluded that the HU-induced N-Cap phenotype consists of an enlargement of the perinuclear ER lumen that likely triggers the clustering of NPCs, which remain functional, in one region of the NE opposite the expanded ER.

**Fig 2:**
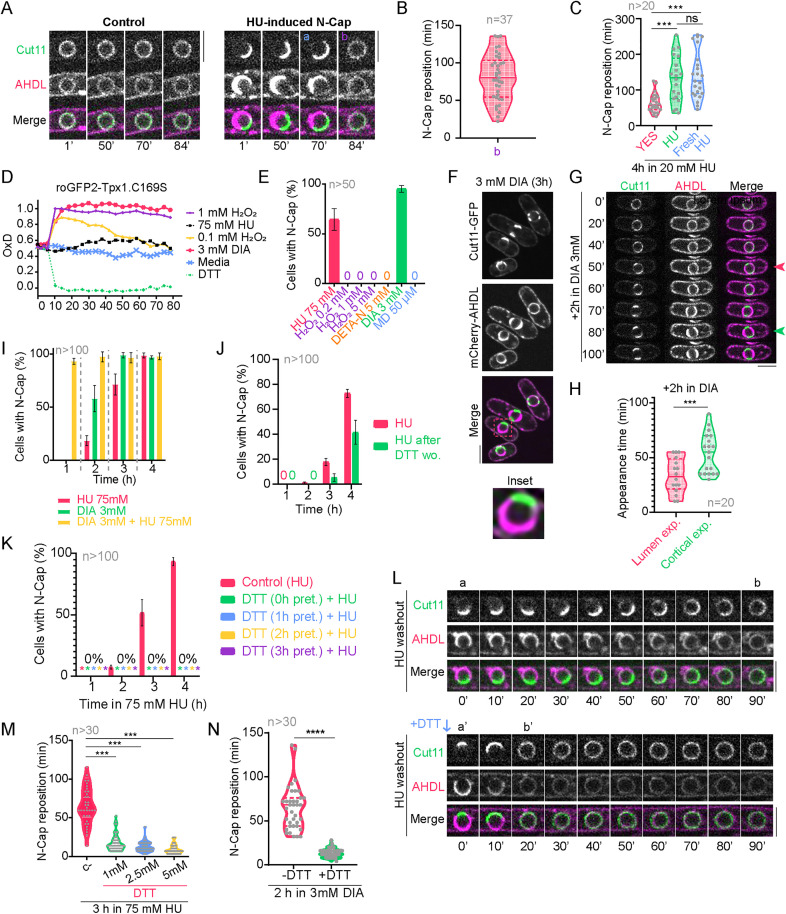
HU-induced ER expansion is reversible and mimicked by thiol stress. **(A)** Confocal microscopy images comparing a nucleus with even NPC and ER distributions, as tagged by Cut11-GFP and mCherry-AHDL respectively (left), and a nucleus with an N-Cap after incubating 3 hours in 75 mM HU (right). ‘a’ marks the last time point before N-Cap redistribution; ‘b’ marks the recovery of the even perinuclear architecture. **(B)** Quantification of the time N-Caps take to recover their isometric distribution (‘b’) along the NE after a 3-hour incubation in 75 mM HU following drug washout. The graph shows a violin plot with the mean ± SD of the 37 cells accounted for in the experiment. **(C)** Quantification N-Cap reposition time after a 4-hour incubation in 20 mM HU. ‘YES’ indicates that HU was washed out of the medium and clean medium was inoculated to the cells; ‘HU’ indicates that the medium remains the same from the start of the incubation with HU; ‘Fresh HU’ indicates that fresh YES media with HU was inoculated to the cells at the same time cells under the ’YES’ category were inoculated with clean medium. Graph shows violin plots with the mean ± SD of the reposition time of at least 20 cells accounted for in each condition. **(D)** H_2_O_2_ production by HU using the biosensor roGFP2-Tpx1.C169S. Quantification of the degree of probe oxidation, or OxD (amount of probe oxidation per 1), of roGFP2-Tpx1.C169S over time. Cultures of strain HM123 carrying plasmid p407.C169S were treated with 1 mM H_2_O_2_ (to achieve maximal probe oxidation, 1), 50 mM DTT (it induces full reduction of the probe, 0), water (control), 0.1 mM H_2_O_2_, 75 mM HU or 3 mM diamide (DIA). The average of three biological replicates is represented. **(E)** Quantification of the incidence of the N-Cap phenotype in 75 mM HU, 0.2 mM H_2_O_2_, 1 mM H_2_O_2_, 5 mM H_2_O_2_, 5 mM DETA-NONOate (DETA-N), 3 mM DIA and 50 μm menadione (MD) after a 3-hour incubation. Apart from HU, only DIA reproduces the N-Cap phenotype. **(F)** Confocal microscopy images of cells treated with 3 mM DIA for 3 hours and an inset of a representative nucleus showing the N-Cap phenotype, as seen with tags Cut11-GFP and mCherry-AHDL. **(G)** Images showing a timelapse of the cellular architecture modification after DIA exposure. The magenta arrow marks N-Cap apparition; the green arrow marks the apparition of cortical ER expansions. **(H)** Quantification of the appearance time of the N-Cap phenotype (‘Lumen exp.’ stands for ‘lumen expansion’) and of the cortical expansions (‘Cortical exp.’). The graph shows violin plots with the mean ± SD of the apparition time of the indicated phenotype of at least 20 cells. **(I)** Quantification of the incidence of the N-Cap phenotype in a population of cells over time in 75 mM HU, 3 mM DIA or a combination of the two. Combining HU and DIA leads to a synergic effect upon N-Cap formation. **(J)** Quantification of the incidence of the N-Cap phenotype in a cell population during a 4-hour treatment with 75 mM HU (‘HU’) and a culture pretreated with 1 mM DTT for 1 hour before DTT washout and then inoculated with 75 mM HU (‘HU after DTT wo.’). **(K)** Quantification of the incidence of the N-Cap phenotype in a cell population during a 4-hour treatment with 75 mM HU (‘Control (HU)’), during a treatment combining 75 mM HU and 1 mM DTT (‘DTT (0h pret.)+HU’), or during the same treatment with a prior pretreatment with 1 mM DTT for 1 hour (‘DTT (1h pret.)+HU’), 2 hours (‘DTT (2h pret.)+HU’) or 3 hours (‘DTT (3h pret.)+HU’). **(L)** Images comparing a timelapse of N-Cap reposition in two representative nuclei after a 3-hour incubation in 75 mM HU, one in which clean YES medium is added (upper panel) and the other with 1 mM DTT added when HU is washed out of the medium (lower panel). Addition of DTT to the medium when washing HU from a culture leads to the rapid redistribution of NPCs and ER, as seen with tags Cut11-GFP and mCherry-AHDL respectively. **(M)** Quantification of the reposition time of N-Caps after HU washout following a 3-hour incubation in 75 mM HU. Higher DTT concentrations cause a faster dispersion of the phenotype. **(N)** Quantification of the reposition time of N-Caps after DIA washout with and without DTT added to the medium after a 2-hour incubation in 3 mM DIA. N-Caps formed in DIA dissolve when the drug is washed out from the medium, and this dissolution occurs faster when adding DTT. All confocal microscopy images are SUM projections of three central Z slices. Scale bars represent 5 microns. Graphs in (B), (L) and (M) show violin plots with the mean ± SD of the reposition time of N-Caps in least 20-30 cells (as indicated in the n) accounted in each condition. Graphs in (E), (I), (J) and (K) show the mean ± SD of two independent repetitions of each experiment, with at least 50-100 cells (as indicated in the n) accounted in each condition. For additional information, consult S2 Fig. Source data for this figure and for S2 Fig can be found in S1 Data.

To determine whether HU-induced N-Caps were a terminal phenotype or a transient response, we induced their formation in cells expressing Cut11-GFP and mCherry-AHDL by incubation in 75 mM HU-containing media for 3 hours. Then, HU was washed out and cells were filmed by confocal microscopy. We found that both the perinuclear ER and NPCs concomitantly recovered their isometrical distribution along the nuclear periphery on average 80.86 ± 31.31 (*n* = 37) minutes following HU washout (Fig 2A, 2B; for results, see S1 Data). Interestingly, if HU remained in the medium for a longer period, or even if the medium was re-inoculated with fresh HU, perinuclear ER and clustered NPCs eventually returned to their normal architecture (Fig 2C), indicating that cells adapt to high doses of the stress caused by HU. We also found that the time needed to restore normal NPC distribution depended on the size of the perinuclear ER lumen (S2A, S2B Fig; for results, see S1 Data). Therefore, our results indicate that the enlargement of the perinuclear ER lumen induced by HU is transitory.

### Perinuclear ER expansion involves INM and ONM/ER separation and requires NE sealing and repair

To characterize HU-induced ER expansion, we tagged several NE components. We observed that the expanded ER membrane contained inner nuclear membrane (INM) proteins Lem2-GFP and GFP-Ima1 (S2C Fig), while INM protein Man1-GFP accumulated coincident with the localization of NPCs (S2D Fig), consistent with recent findings [36]. Next, we performed transmission electron microscopy (TEM) of HU-treated cells in the conditions of maximum ER expansion (incubation for 4 hours) along with wild-type untreated cells. TEM images confirmed that while the INM and the outer nuclear membrane (ONM) remain in very close proximity in control conditions, a clear phenotype of INM and ONM separation was observed under perinuclear ER lumen expansion (S2E, S2F Fig). Thus, both live cell images of GFP-tagged INM proteins and TEM images revealed that the perinuclear ER expansion involves separation of the INM and ONM, which, in turn, constricts NPCs in the opposite region of the NE.

The INM and ONM/ER form a unique, continuous NE membrane in which NPCs are embedded [37,38]. NE sealing and repair mechanisms rely on the ESCRT-III complex, composed of Cmp7, Vps4, and nuclear specific adaptor Lem2, among other elements. This complex assists with the elimination of aged and damaged NPCs, and repairs and seals discontinuities on the NE [39,40]. In addition, Lem2 functions as a barrier to maintain nuclear membrane identity regulating membrane flow between NE and ER [41,42]. We speculated that, if perinuclear ER expansion results from INM-ONM/ER separation, then perturbing the NE-ER boundary and NE sealing and repair mechanisms might interfere with ER expansion. As expected, the addition of HU to cells deleted for *lem2*, *cmp7* or *vps4* resulted in perinuclear ER fragmentation (S2G, S2H Fig; for results, see S1 Data). These data show that perinuclear ER lumen expansion requires ER-NE remodeling, as well as NE sealing and repair mechanisms, to ensure that this expansion occurs while maintaining nuclear and ER compartmentalization and integrity.

### HU-induced perinuclear ER expansion is mimicked by thiol stress and is conserved in eukaryotic cells

It has been shown that HU treatment produces ROS *in vivo*, including H_2_O_2_ and nitric oxide (NO) [43,44]. We confirmed that HU induces oxidative stress in fission yeast, as the biosensor roGFP2-Tpx1.C169S, which is finetuned for detecting H_2_O_2_ fluctuations [45], showed a clear oxidation in the cytosol during HU treatment, similar to oxidants diamide (DIA) or H_2_O_2_, although H_2_O_2_ and specially DIA oxidize the sensor faster and more effectively (Fig 2D; for results, see S1 Data). However, treatment with increasing concentrations of H_2_O_2_ did not induce N-Cap formation (Fig 2E). Addition of DETA-NONOate, a compound that generates NO, did not produce any observable ER phenotype either (Fig 2E). We further tested if treatment with other oxidants, such as menadione (MD), which generates superoxide [46], and DIA, which is a membrane-permeable specific thiol oxidant [47,48], resulted in N-Cap formation. Whereas MD did not produce any noticeable N-Cap phenotype (Fig 2E), DIA mimicked the effect of HU and led to the emergence of N-Caps that were indistinguishable from those observed during HU treatment (Fig 2E, 2F). Similarly to HU, both NPC clustering and perinuclear ER lumen expansion emerged concomitantly during DIA treatment, and DIA also induced cortical ER expansion at longer exposition times (Fig 2G, 2H). Furthermore, DIA and HU acted synergistically, vastly increasing the incidence of N-Caps when combined (Fig 2I).

DIA has been shown to oxidize GSH to GSSG, depleting reduced glutathione in the cell [47] and indirectly promoting disulfide bond formation and glutathionylation in proteins containing thiol groups (-SH), which is known as thiol or disulfide stress [49,50]. Conversely, dithiothreitol (DTT) is a strong reductant that turns oxidized cysteines back to their reduced state, breaks disulfide bonds and reverts protein glutathionylation [51,52]. Thus, we hypothesized that DTT treatment might prevent and/or revert N-Cap formation if this HU-induced phenotype was due to hyperoxidation of thiol groups, as suggested by the reproduction of this phenotype in DIA. Consistently, when cells were pretreated with DTT and either washed out or left in the media before adding HU, the appearance of N-Caps was significantly reduced or avoided (Fig 2J, 2K; for results, see S1 Data). Moreover, DTT addition to HU- or DIA-treated cells already displaying N-Caps reverted this phenotype with accelerated kinetics than simple drug washout (Fig 2L, 2N). These data suggest that perinuclear ER expansion induced by HU or DIA is caused by alterations in the redox folding capacity caused by these oxidants, which is consistent with a recent publication [25].

Next, we addressed whether HU-induced NPC clustering and ER expansion were evolutionarily conserved. We first tested if NPC clustering was observed in the budding yeast *S. cerevisiae* and, indeed, in cells expressing NUP84-mCherry, NPC clusters were noticed after a 3-hour incubation in 100 mM HU (S3A Fig), and the dynamic NPC reposition after HU washout was also recapitulated (S3B Fig), indicating that this phenotype is not specific to *S. pombe*. We further confirmed the conservation of the ER expansion phenotype in mammalian cells by using HT1080 fibrosarcoma cells and incubating them with 200 µM and 1 mM HU (S3C Fig) and with 50 µM DIA (S3D Fig; for results, see S1 Data). ER was stained with the ER-ID Red Assay Kit and its total intensity measured following exposure to both drugs, as well as after exposure to DTT as a negative control. Importantly, both HU and DIA treatments led to a clear increase in ER total intensity, which is indicative of an increment in ER size [53,54], while DTT treatment reduced its intensity. This shows that HU- and DIA-induced ER expansion is conserved in eukaryotic cells.

### ER expansion correlates with a block in the secretory pathway

**Fig 3:**
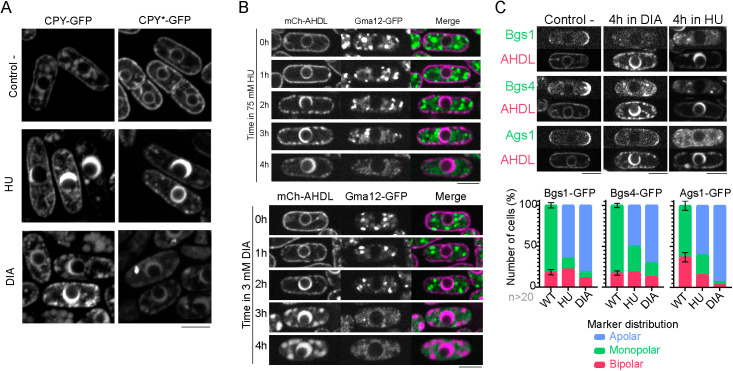
Perinuclear ER expansion disrupts vesicular transport. **(A)** Images of representative cells expressing wildtype CPY-GFP (upper panels) and the mutant allele CPY*-GFP (below upper panels in untreated control conditions, and after a 4-hour treatment with either 75 mM HU or 3 mM DIA. **(B)** Images showing Golgi morphology, marked by Gma12-GFP, over time during exposure to either 75 mM HU (upper panels) or 3 mM DIA (lower panels). N-Cap presence was also assessed with mCherry-AHDL tagging of the luminal ER. The images show how the vesicular morphology of the Golgi apparatus is lost over time. **(C)** Images of representative cells comparing exocytosis markers Bgs1-GFP, Bgs4-GFP and Ags1-GFP against the luminal ER tag mCherry-AHDL, in control conditions and after a 4-hour incubation in either 3 mM DIA or 75 mM HU. These images show how all three exocytosis markers relocate from the cell tips to the lateral cortex and the cytoplasm in most of the population. All confocal microscopy images are SUM projections of three central Z slices. Scale bars represent 5 microns. Source data for this figure can be found in S1 Data.

As ER expansion induced by HU and DIA might be caused by an accumulation of unfolded luminal proteins, we analyzed the localization of the well-characterized luminal marker Sc. Carboxypeptidase Y (CPY) [55,56]. ScCPY is a soluble vacuolar protein that is synthesized as a pre-pro-peptide, imported into the ER, where the pre-(signal) sequence is cleaved off, then transits through the Golgi towards the vacuole, where the pro-sequence is cleaved giving the mature and functional enzyme. A mutated allele of ScCPY (ScCPY*, Gly255Arg) is imported into the ER, but due to its defective folding, it never reaches the vacuole; instead, it is retained in the ER, retrotranslocated, and degraded through the luminal ERAD pathway (ERAD-L) [56–58]. We first analyzed wild-type ScCPY-GFP localization during HU or DIA treatment. In control untreated conditions, as described, ScCPY-GFP localized to vacuoles, which is indicative of proper protein synthesis and ER and Golgi transit, folding, and maturation. However, both HU and DIA treatments resulted in its accumulation at the enlarged ER lumen and its deprivation from vacuoles (Fig 3A). ScCPY*-GFP localized at the perinuclear and cortical ER, indicative of defective exit from this compartment in control conditions, and HU and DIA treatments also resulted in an increase of ScCPY*-GFP at the enlarged ER lumen (Fig 3A), which suggests defective retrotranslocation and degradation. Our results show that both the functional and the mutant CPY accumulate at the ER lumen in the presence of HU or DIA and are consistent with recent findings showing that HU induces oxidation of cysteines and formation of disulfide bonds in ScCPY*, and propose that HU induces aberrant disulfide bond formation likely in a variety of ER luminal proteins, which would interfere with ER retrotranslocation and their degradation by ERAD-L [25].

The ER and the Golgi apparatus maintain equilibrium through bidirectional transport; therefore, we reasoned that the effect of HU or DIA on the ER might directly impinge on Golgi functionality and secretion [59]. By following the Golgi marker Gma12-GFP [60], we found that both drugs impacted on the Golgi apparatus morphology and distribution, reducing its signal the longer cells were exposed to the drugs (Fig 3B). Consistently, polarized secretion of glucan synthases Bgs1-GFP, Bgs4-GFP, and Ags1-GFP [61–64] was impaired during HU or DIA treatments (Fig 3C; for results, see S1 Data). Therefore, our results show that HU and DIA treatments disrupt the functionality of the secretory system.

### Chaperone Bip1 accumulates at the enlarged ER lumen while cytoplasm chaperones aggregate in the cytoplasm in HU- or DIA-treated cells

**Fig 4:**
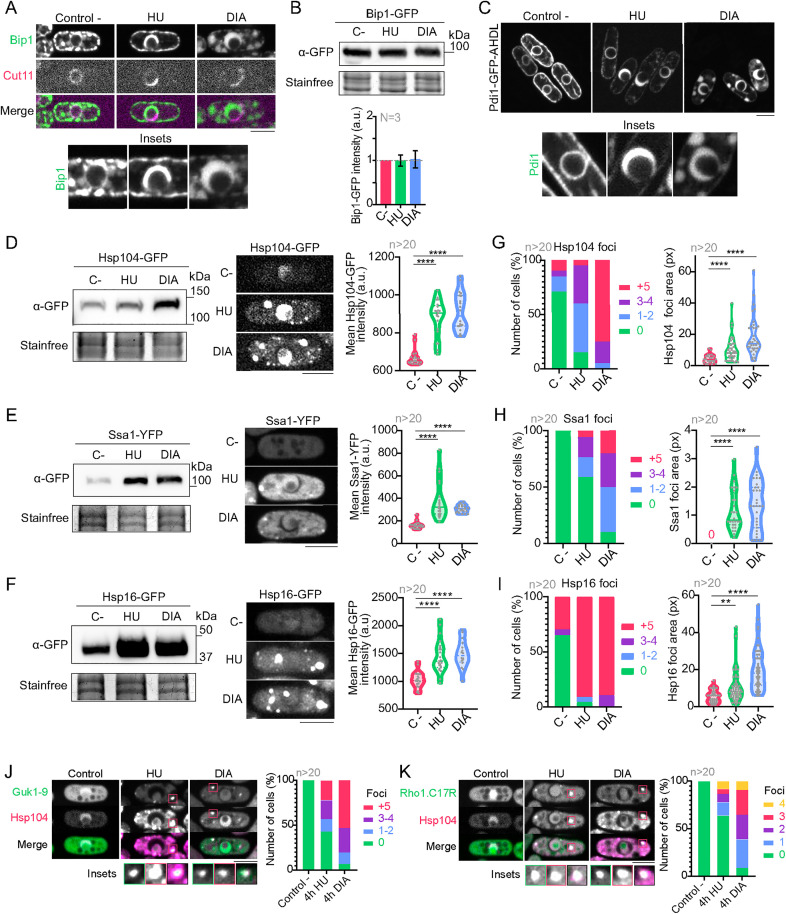
HU and DIA induce cytoplasmic protein and chaperone aggregation. **(A)** Confocal microscopy images showing the distribution of luminal ER chaperone Bip1-GFP in untreated conditions, where it decorates both the perinuclear and cortical ER, and after treatments with 75 mM HU or 3 mM DIA for 4 hours. Bip1-GFP localizes to the expanded perinuclear ER lumen in both conditions, depriving the cortical ER of some of its fluorescence. Panels below show insets of the perinuclear region of each cell. **(B)** western blot showing the total amount of Bip1-GFP, as tagged by an anti-GFP antibody, in control untreated conditions, and after a 3-hour incubation either in 75 mM HU or 3 mM DIA. Bip1-GFP amount remains constant in both treatments as compared with the control. Stain-free lanes are shown as loading control in the lower panels. The graph on the right shows the mean of Bip1-GFP intensity in three independent repetitions of the western blot, normalized to the intensity of the untreated control. **(C)** Images of representative cells expressing Pdi1-sfGFP-AHDL in control conditions and after a 4-hour incubation either in 75 mM HU or 3 mM DIA. Panels below show insets of the perinuclear region of each cell. **(D-F) Left:** western blot showing the total amount of Hsp104-GFP (D), Ssa1-YFP (E) or Hsp16-GFP (F), as tagged by an anti-GFP antibody, in control untreated conditions, and after a 3-hour incubation either in 75 mM HU or 3 mM DIA. All three chaperones increment their total amount in both oxidants with respect to untreated cells. Stain-free lanes are shown as loading control in the lower panels. **Center:** Confocal microscopy images of representative cells in control conditions and after 75 mM HU and 3 mM DIA treatments in strains expressing chaperones Hsp104-GFP (D), Ssa1-YFP (E) or Hsp16-GFP (F). In the case of Hsp104-GFP and Hsp16-GFP, cells were incubated for 3 hours in each drug, while cells expressing Ssa1-YFP were incubated for 4 hours. **Right:** Quantification of the total mean intensity of each of the forementioned chaperones in the conditions previously addressed. Graphs of fluorescence intensity (measured in arbitrary units) show violin plots with their respective mean and SD, where at least 20 cells (n) were accounted for in each condition. **(G-I) Left:** Quantifications of the number of cytoplasmic foci per cell of Hsp104-GFP (G), Ssa1-YFP (H) or Hsp16-GFP (I) in the conditions addressed in (D-F). Graphs are normalized to the total number of cells per condition, and >20 cells were analyzed for each. **Right:** Quantifications of the foci area of the cytoplasmic aggregates noticed with the respective chaperones in the conditions previously addressed. Graphs show violin plots with their respective mean and SD of the foci area (measured in pixels), where at least 20 cells (n) where accounted for in each condition. **(J-K) Left:** Representative cells expressing aggregation markers sty1::guk1-9-GFP (J) and sty1::rho1.C17R-GFP (K) coexpressed with Hsp104-RFP, showing foci after a 4-hour incubation in either 75 mM HU or 3 mM DIA. Insets below highlight colocalizations. **Right:** Quantification of the number of foci of each of the forementioned aggregation markers in control conditions and after incubation with the two drugs. Graphs are normalized to the total number of cells per condition, and >20 cells were analyzed for each. Confocal microscopy images in (A) are SUM projections of three central Z slices, while confocal microscopy images in (C), (D), (E), (I) and (J) are SUM projections of 18 Z slices. Scale bars represent 5 μm. For additional information, consult S4 Fig. Source data for this figure and for S4 Fig can be found in S1 Data. Uncropped western blot images can be found in S1 Raw Images.

Bip1 is a Hsp70 chaperone that localizes in the ER luminal space to assist with the folding and translocation of proteins [65–69]. Additionally, a disaggregase activity of mammalian BiP has been recently reported on ER protein aggregates [70]. Thus, we analyzed the localization of Bip1-GFP in untreated cells and in cells treated with HU or DIA. In untreated cells, Bip1-GFP localized at the thin perinuclear ER luminal space, as well as in a dotted pattern along the cortical ER (Fig 4A). However, upon HU or DIA treatments, Bip1-GFP cortical foci decreased their number and intensity concomitantly with an increase of Bip1-GFP signal at the expanded perinuclear ER (Fig 4A), whereas the protein levels of Bip1 did not significantly change (Fig 4B). This suggests that most of the cellular pool of Bip1 relocates from the cortical ER to the expanded perinuclear ER lumen both in HU or DIA treatments. We further explored Bip1-GFP dynamics by FRAP. We found that Bip1-GFP signal recovers after photobleaching the perinuclear ER in HU-treated cells that had not yet expanded the ER, showing that Bip1 is dynamic between cortical and perinuclear ER in these conditions. However, Bip1-GFP signal did not recover after photobleaching the whole expanded ER in cells that had fully developed this phenotype, whereas it did recover when only half of the expanded region was bleached (S4A, S4B Fig; for results, see S1 Data). This suggests that Bip1-GFP is mobile within the expanded perinuclear ER but cannot freely diffuse between the cortical and the perinuclear ER once the N-Cap is formed. Our results agree with a recent study in *S. cerevisiae* that demonstrates biochemically the recognition of ScCPY* by Kar2/Bip upon HU treatment [25]. Thus, HU and DIA treatments result in protein misfolding in the perinuclear ER lumen, which immobilizes free Bip1.

We also analyzed the effect of HU or DIA treatments on the localization of the ER disulfide isomerase Pdi1-GFP, another luminal protein whose cysteines oxidize to disulfides upon HU treatment [25], and found that it behaved similar to Bip1-GFP (Fig 4C). Furthermore, we analyzed whether DIA and HU were also engaging cytoplasmic chaperones. Indeed, we found that both treatments led to an increase in the levels of Hsp70/Ssa1-YFP, Hsp16-GFP, and the disaggregase Hsp104-GFP and their accumulation in cytoplasmic foci, suggesting defective folding in this compartment (Fig 4D–4F; for results, see S1 Data). These foci increased their number and size over time and were always more prominent in DIA (Fig 4G–4I). To test whether cytoplasmic foci represent aggregates of misfolded proteins, we analyzed the behavior of mutants in guanylate kinase (Guk1–9-GFP) and GTPase Rho (Rho1.C17R-GFP) that have been previously used as reporters of misfolding upon heat stress [71,72]. Both, Guk1-9-GFP and Rho1.C17R-GFP accumulated in cytoplasmic foci that colocalized with Hsp104-mRFP foci upon DIA and HU treatments (Fig 4J, 4K). We also tested if these foci contained Vgl1-GFP, which is one of the main components of heat shock-induced stress granules in *S. pombe* [73]. However, we found that HU- or DIA-induced foci lacked this stress granule marker, and indeed Vgl1 did not form foci in response to these treatments (S4C Fig). Therefore, HU or DIA aggregates differ from the canonical heat stress induced granules. We also determined that these aggregates were not bound to membranes, as these Hsp104-aggregates did not colocalize with membrane proteins such as Ish1-mScarlet (S4D Fig), ER transmembrane proteins Rtn1-GFP and Yop1-GFP (S4E, S4F Fig), nor with Gma12-GFP that marks the Golgi apparatus (S4G Fig), suggesting that cytoplasmic protein misfolding arises independently of protein misfolding in the ER lumen.

### Inhibition of protein synthesis with puromycin suppresses ER expansion but exacerbates cytoplasmic aggregation

**Fig 5:**
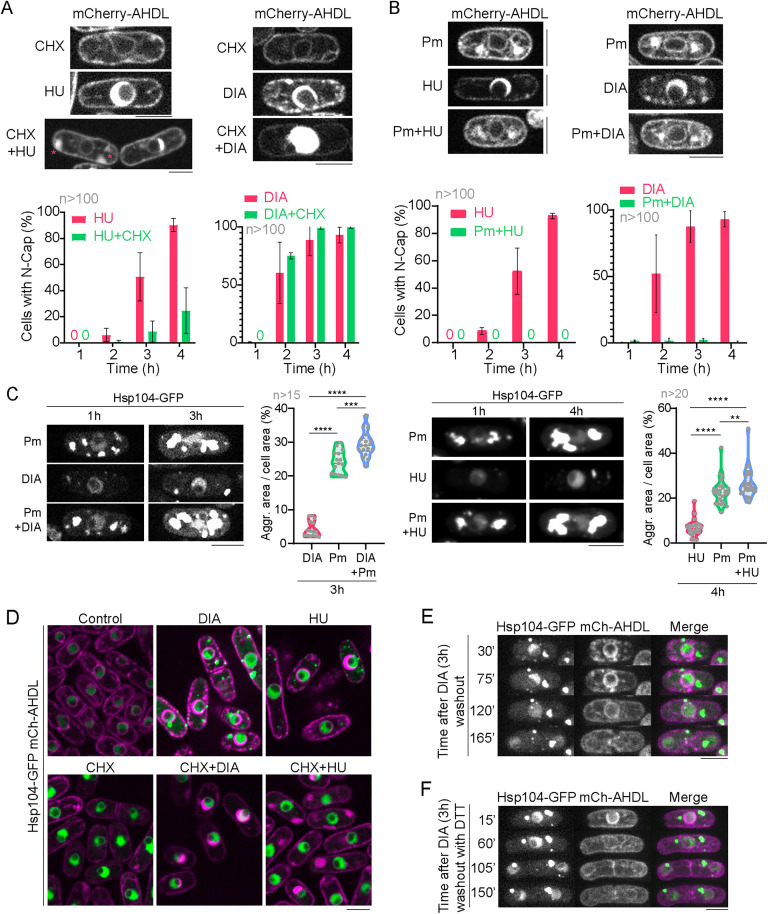
ER expansion and cytoplasmic aggregates are linked to de novo protein synthesis. **(A) Upper panels:** Images of representative cells expressing luminal ER tag mCherry-AHDL exposed to 75 mM HU, 3 mM DIA, 100 μg/mL cycloheximide (CHX) or a combination of the drugs, as indicated, for 4 hours. Asterisks in CHX + HU indicate cortical ER expansion. **Lower panels:** Graphs showing N-Cap incidence in cells exposed to the addressed drugs. **(B) Upper panels:** Images of representative cells expressing mCherry-AHDL exposed to 75 mM HU, 3 mM DIA, 5 mM puromycin (Pm) or a combination of the drugs, as indicated, for 4 hours. **Lower panels:** Graphs showing N-Cap incidence in cells exposed to the addressed drugs. **(C) Left:** Images of cells expressing Hsp104-GFP after a 1-hour or 3-hour incubation in 5 mM Pm, 3 mM DIA, or a combination of both drugs. **Left, graph:** Quantification of the aggregated area, as tagged with Hsp104-GFP, normalized to the total cell area of cells after a 3 hour treatment in either 5 mM Pm, 3 mM DIA, or a combination of both drugs. **Right:** Confocal microscopy images of cells expressing Hsp104-GFP after a 1-hour or 4-hour incubation in 5 mM Pm, 75 mM HU, or a combination of both drugs. **Right, graph:** Quantification of the aggregated area, as tagged with Hsp104-GFP, normalized to the total cell area of cells after a 4-hour treatment in either 5 mM Pm, 75 mM HU, or a combination of both drugs. The graphs show violin plots with their respective mean and SD, with >15-20 cells (n) accounted for in each condition. **(D)** Confocal microscopy images of cells expressing Hsp104-GFP (green) and mCherry-AHDL (magenta) after a 3-hour incubation in either control conditions, 100 μg/mL CHX, 3 mM DIA, 75 mM HU, DIA and CHX, or HU and CHX. Hsp104-GFP aggregates are detected only in DIA and HU treatments. Images are SUM projections of 3 central Z slices. **(E)** Timelapse showing that cytoplasmic aggregates observed in Hsp104-GFP after DIA treatment, in cells also expressing mCherry-AHDL, do not dissolve over time. **(F)** Timelapse showing that cytoplasmic aggregates observed in Hsp104-GFP after DIA treatment, in cells also expressing mCherry-AHDL, are neither dissolved by 1 mM DTT addition. Confocal microscopy images in (C), (E) and (F) are SUM projections of 18 Z slices. Scale bars represent 5 μm. Graphs in (A) and (B) show the mean ± SD of at least 100 representative cells in each condition in three independent repetitions of the experiment. For additional information, consult S5 Fig. Source data for this figure and for S5 Fig can be found in S1 Data.

As protein misfolding caused by HU or DIA might be especially critical during protein synthesis, we tested whether inhibiting de novo protein synthesis with cycloheximide (CHX) affected ER expansion or cytoplasmic aggregation. For that, cells were treated with either HU or DIA, and with or without 100 μg/ml CHX, a concentration that fully blocks protein synthesis in *S. pombe* [74]. Whereas treating fission yeast with HU alone resulted in 90.33 ± 5.03% of cells (n > 100) with expanded ER (Fig 5A, left; for results, see S1 Data), combining HU + CHX resulted in 24.67 ± 17.50% (n > 100) cells with expanded ER after a 4-hour incubation. However, these cells did show cortical ER expansions (Fig 5A, asterisks). When DIA was combined with CHX, perinuclear ER lumen expansion was exacerbated compared to cells incubated with DIA alone (Fig 5A, right). CHX blocks translation elongation by ‘freezing’ ribosomes and leaving incomplete nascent peptides attached to them [75,76], which might exacerbate the ER expansion phenotype as these peptides are cotranslationally translocated into the ER. Meanwhile, puromycin (Pm), another drug that also inhibits protein synthesis, blocks elongation and liberates nascent peptides and disassembled ribosomes into the cytoplasm for a new round of unproductive translation [77,78] in combination with either HU or DIA resulted in the total suppression of perinuclear ER expansion (Fig 5B) and the appearance of cytoplasmic Hsp104-GFP foci (Fig 5C). Indeed, Pm treatment alone induced the appearance of cytoplasmic Hsp104-GFP foci, and combining DIA or HU and Pm exacerbated this phenotype (Fig 5C). As releasing nascent peptides from ribosomes with Pm fully suppresses perinuclear ER expansion, while blocking their synthesis at ribosomes with CHX exacerbates this phenotype in the perinuclear (DIA) or in the cortical (HU) ER, while avoiding the formation of cytoplasmic aggregates (Fig 5D), these results suggest that HU and DIA interfere with peptide folding during their synthesis. We also tested protein synthesis inhibitor anisomycin, which, similar to CHX, blocks protein synthesis and stabilizes polysomes [79], and found that this drug in combination with HU or DIA mimicked CHX behavior, exacerbating perinuclear ER expansion in combination with DIA and cortical ER expansion when combined with HU (S5A, S5B Fig; for results, see S1 Data). Interestingly, HU- or DIA-induced cytoplasmic aggregates are not reversible by drug washout (Fig 5E) nor eliminated upon DTT treatment (Fig 5F).

### HU and DIA elicit a transcriptional response that differs from canonical unfolded protein response

Whereas interfering with protein folding in the ER by tunicamycin (Tn), a drug that impinges on protein glycosylation, or by DTT treatment which activates the UPR, oxidative stress can potentially impact on protein folding without activating the canonical UPR pathway [80,81]. The major UPR initiator is IRE1, an ER transmembrane kinase endoribonuclease that functions as stress sensor in the ER. When activated by the presence of unfolded proteins, Ire1 performs a non-conventional splicing on Hac1/XBP1 mRNA, which results in the production of UPR transcriptional activators [8,9]. However, *S. pombe* lacks a Hac1/XBP1 ortholog and a UPR-dependent transcriptional response. *S. pombe* Ire1 is responsible for the selective decay of mRNAs encoding ER proteins, particularly proteins involved in lipid and sterol metabolism, thus decreasing the ER load during stress, in a pathway called RIDD (regulated Ire1-dependent decay). In fission yeast, *bip1* mRNA is recognized and cleaved by Ire1 but remains stable, escaping decay and increasing its translation [82,83]. We tested whether HU- or DIA-induced ER expansion was dependent on the UPR. To this end, we treated cells with DTT or Tn and found that these treatments led to a phenotype of cortical ER expansion as described in *S. cerevisiae* [10,84]; however, neither drug resulted in perinuclear ER expansion (S6A Fig). Moreover, *ire1Δ* cells expanded the perinuclear ER under DIA treatment with the same frequency and timing as wild-type cells (S6B Fig; for results, see S1 Data), which shows that Ire1 is not required for this response. Consistently, whereas *ire1Δ* cells were hypersensitive to DTT, they showed a sensitivity to DIA similar to wild-type cells (S6C Fig). Thus, these data indicate that ER expansion is independent of Ire1-mediated UPR, as canonical ER stressors do not replicate the phenotype and deleting Ire1 does not prevent perinuclear ER expansion. Accordingly, the recent study by Takano *and colleagues* demonstrates that HU itself does not induce the UPR in the budding yeast [25].

We then decided to characterize the transcriptomic changes associated with ER expansion observed under HU or DIA exposure. For that, we performed an RNA-Seq analysis from cells exposed to each drug at two time points (prior or during ER expansion; see Methods) as well as untreated wild-type cells. The complete dataset is publicly available in *Gene Expression Omnibus* under the accession number GSE309439, while the processed data is available in S1 Data. The principal component analysis of the five conditions analyzed pointed to well-differentiated transcriptomic programs in DIA and HU (S6D Fig). This was further confirmed in the expression profiles of the upregulated and downregulated genes from both conditions relative to untreated cells (S6E Fig), with waves of overexpression and repression that clearly showed different patterns between conditions. Notwithstanding, there were 97 upregulated genes (LFC ≥ 1, *p*-value ≤ 0.05) common to HU and DIA treatments when both time points were pooled together, some of which are highlighted in S6H Fig, and 24 that are common to all four individual conditions (S6G–S6I Fig). The functional enrichments for the biological processes (S6H Fig) and molecular functions (S6I Fig) of the common transcriptomic signature of both drugs show an upregulation of oxidative stress genes and genes related to iron ion homeostasis. Importantly, chaperones such as Hsp70-Ssa1 and Hsp16, and disaggregase Hsp104 were upregulated relative to control cells, which was demonstrated previously (Fig 4C–4H).

We further compared our transcriptomic data with that obtained by Kimmig *and colleagues* [82] from DTT-treated *S. pombe* cells. When we compared the genes downregulated in our conditions with the ones described by Kimmig *and colleagues* (FC ≤ −1), we found no similarities between HU treatment and UPR-induced downregulation, and only three genes (*ist2*, *efn1*, and *xpa1*), all of which encode for transmembrane proteins, were common with DIA treatment. These results show that HU- or DIA-induced transcriptomic programs are different from DTT-induced UPR, as they do not heavily rely on mRNA decay and favor gene overexpression. Interestingly, we found similarities between genes that were shown to be upregulated more than 2-fold by DTT in Kimmig *and colleagues*, and HU and DIA conditions. Specifically, we found eight common upregulated genes in the three conditions (*frp1*, *plr1*, *SPCC663.08c*, *srx1*, *gst2*, *str3*, *caf5*, and *hsp16*), which are mostly involved in oxidoreduction processes, further suggesting oxidative and folding stress. All this considered, the transcriptome analysis under both HU- or DIA-treatments supports that ER expansion is caused by oxidative stress and differs from a canonical UPR response.

### ER expansion caused by HU or DIA is GSH-dependent and both oxidants induce ectopic protein glutathionylation

**Fig 6:**
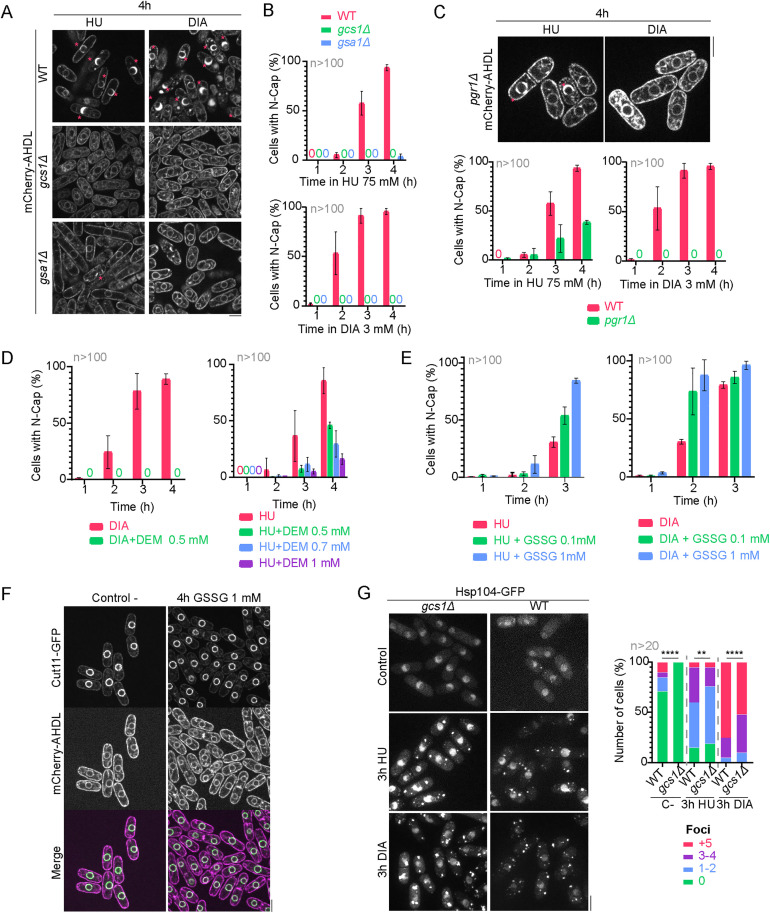
ER expansion is GSH-dependent. **(A)** Confocal microscopy images of wild-type, *gcs1Δ* and *gsa1Δ* strains expressing mCherry-AHDL after a 4-hour incubation in either 3 mM DIA or 75 mM HU. Magenta asterisks indicate cells with N-Caps. **(B)** Quantification of the incidence of the N-Cap phenotype in the previously addressed strains during 75 mM HU (left graph) or 3 mM DIA (right graph) treatments. **(C) Upper panels**: Confocal microscopy images of wild-type and *pgr1Δ* strains expressing mCherry-AHDL after a 4-hour incubation in either 3 mM DIA or 75 mM HU. Asterisks indicate cells with N-Caps. *pgr1Δ* mutants only develop N-Caps under HU exposure. **Lower panels:** Quantification of the incidence of the N-Cap phenotype in wild-type and *pgr1Δ* strains during 75 mM HU (left graph) or 3 mM DIA (right graph) treatments. **(D)** Quantification of the incidence of the N-Cap phenotype in a wild-type strain treated with 3 mM DIA or DIA combined with 0.5 mM DEM (left graph), and with 75 mM HU or HU combined with 0.5, 0.7 mM or 1 mM of DEM (right graph). **(E)** Quantification of the incidence of the N-Cap phenotype in a wild-type strain treated with 75 mM HU or HU combined with 0.1 mM or 1 mM of GSSG (left graph), and with 3 mM DIA or DIA combined with 0.1 mM or 1 mM of GSSG (left graph). **(F)** Confocal microscopy images of wild-type cells expressing Cut11-GFP and mCherry-AHDL in untreated conditions and after a 4-hour incubation in the presence of 1 mM GSSG. The internal morphology of the cells remains unaltered after exposure to GSSG. **(G) Upper panels:** Confocal microscopy images of wild-type and *gcs1Δ* strains expressing Hsp104-GFP after a 3-hour incubation in either 3 mM DIA or 75 mM HU. Images are SUM projections of three central Z slices. Scale bars represent 5 microns. **Lower panels:** Quantification of the number of Hsp104-GFP foci observed in a wild-type and a *gcs1Δ* strain after a 3-hour treatment with either HU or DIA. The graph is normalized to the total number of cells per condition, and >20 cells (n) were analyzed for each. Confocal microscopy images in (A), (C) and (F) are SUM projections of three central Z slices, while confocal microscopy images in (F) are SUM projections of 18 Z slices. Scale bars represent 5 μm. Graphs in (B), (C), (D) and (E) show the mean ± SD of two independent repetitions of the experiment, and in each repetition at least 100 cells were accounted for each condition. Source data for this figure can be found in S1 Data.

GSH is an antioxidant non-protein thiol that plays a central role in protecting against oxidative stress. Moreover, it has been previously shown that DIA binds and sequesters GSH and induces ectopic protein glutathionylation [47,85]. The absence of GSH upon its sequestration by DIA might impair the vigilance mechanisms that correct, along with PDIs and oxidoreductases, non-native disulfide bonds in proteins during their folding in the ER [86–88]. Both deregulated glutathionylation and non-native disulfide bond formation lead to protein misfolding [89–92]. To test whether GSH plays a role in ER expansion, we manipulated intracellular GSH levels both genetically and chemically. First, we used strains with a deletion of the *gcs1* gene, which encodes glutamyl cysteine ligase, enzyme that catalyzes the rate-limiting initial step in GSH synthesis (union of L-cysteine and L-glutamate into L-γ-glutamylcysteine), and a deletion of the *gsa1* gene, which encodes glutathione synthase, enzyme that catalyzes the final step of GSH biosynthesis (addition of glycine to L-γ-glutamylcysteine) [93,94], and exposed them to DIA or HU (Fig 6A, 6B; for results, see S1 Data). Importantly, we found that perinuclear ER expansion was suppressed in the absence of Gcs1, and the *gsa1Δ* mutant strain also fully suppressed N-Cap appearance in DIA and significantly reduced its frequency in HU. A third gene involved in the GSH/GSSG equilibrium is *pgr1*, which encodes the glutathione reductase that ensures that oxidized GSSG returns to its reduced state. Consistent, *pgr1Δ* also fully prevented ER expansion under DIA treatment, while the incidence of the HU-induced ER expansion phenotype was significantly reduced in this mutant (Fig 6C).

Diethyl maleate (DEM), a compound that chemically depletes the GSH cellular pool [95], also prevented ER expansion in both HU and DIA (Fig 6D). Furthermore, when cells were exposed to either HU or DIA in the presence of extracellular GSSG, the phenotype of ER expansion was temporally advanced and significantly increased its incidence (Fig 6E). However, addition of GSSG alone does not induce ER expansion (Fig 6F), indicating that the decrease in the GSH/GSSG ratio is not the trigger of the phenotype. We also determined that deletion of *gcs1* did not suppress the accumulation of Hsp104-GFP in cytoplasmic foci (Fig 6G), which supports that cytoplasmic protein aggregates induced by HU and DIA arise from an imbalance in cytoplasmic proteostasis and are not a direct consequence of HU or DIA impact upon ER redox protein folding.

DIA forms an intermediate with GSH, which can lead to the transfer of GSH to reduced sulfhydryl groups in proteins, inducing protein glutathionylation [47,85]. As the phenotype induced by HU is similar to that of DIA, we hypothesized that HU may also induce protein glutathionylation. To test this, we determined peptide glutathionylation in wild-type cells treated with DIA and HU by mass spectrometry. The results showed that both drugs’ treatments led to an increase in glutathionylation of cytoplasmic proteins compared to untreated conditions (S1–S4 Tables). Among the proteins that are glutathionylated by both DIA and HU (S5 Table), there are several directly involved in protein synthesis: four ribosomal proteins (Rps2, Rpl11, Rpl3401, Rpl3402) and two translation elongation factors (Tef1, Tef2) as well as Hsp90. Thus, our results show that HU induces oxidative stress and protein glutathionylation, which complements a recent study showing that HU promotes disulfide formation in cysteines in ER proteins [25].

In summary, our results show that HU tampers with the cellular redox folding capability, both in the ER and the cytoplasm, which is consistent with recent findings that demonstrate that HU modulates thiol-disulfide homeostasis [25].

## Discussion

Oxidative stress can disrupt the proper folding of proteins in the ER and trigger an ER stress response, a process involved in the development and progression of various pathological conditions [96–98]. This work shows that HU causes an expansion of the perinuclear ER lumen that accumulates ER chaperones Bip1 and Pdi1, as well as luminal proteins such as vacuolar CPY or misfolded CPY*, which otherwise is retrotranslocated and degraded by the ERAD-L pathway [56], suggesting that HU interferes with protein folding and transit out of the ER. Furthermore, HU treatment disrupts Golgi morphology and impairs the polarized localization of cell wall glucanases, indicating that these treatments interfere with proper functioning of the secretory system. These effects of HU are independent of RNR inhibition or S-phase arrest, are recapitulated by the thiol stress inducer DIA, and prevented and suppressed by reducing agent DTT, pointing to thiol stress as the main cause of these phenotypes.

Our results are consistent with and complement a recent study [25] showing biochemically that HU selectively inhibits the ERAD-L pathway without affecting the other ERAD pathways in *S. cerevisiae* and independently of its known effect of RNR inhibition. Takano *and colleagues* show that HU promotes ectopic disulfide bond formation in ER luminal proteins such as Pdi1 or CPY*, impairing their retrotranslocation during ERAD-L. This sets a precedent showing that HU tampers with thiol homeostasis, which complements our observations and positions HU as a disulfide-inducing compound able to target proteins in the ER. Our results align with previous studies showing that ER stress can result in expansion of ER membranes and luminal volume to increase its protein folding capacity, preventing unfolded or misfolded proteins from forming aggregates [10,84,99–104]. ER expansion induces structural changes at the nuclear membrane, including INM-ONM/ER separation, which is likely the cause of NPC clustering. Although clustered NPCs remain functional and ER expansion is transient, these changes resemble features seen in aged cells and in nuclear envelopathies and laminopathies, diseases related to mutations in genes encoding nuclear envelope proteins, especially lamins [105–109]. Although yeast lacks nuclear lamina, it still exhibits a high level of nuclear organization that relies on INM proteins and associated complexes [110,111]. We speculate that ER expansion in eukaryotes with an unperturbed nuclear lamina would not lead to NPC clustering due to the structural support it provides, but the effects might differ in cells with disrupted lamina.

We show that HU-induced ER stress in *S. pombe* does not result in Ire1-mediated UPR activation, and instead shows a unique transcriptomic signature characterized by the upregulation of oxidative stress response genes, genes involved in ion homeostasis, and genes encoding chaperones. This atypical ER stress response is not unprecedented; in *S. cerevisiae*, HU-induced ERAD-L inhibition has also been reported to occur independently of UPR activation [25], and non-canonical UPR-independent ER stress responses have been described in other systems, involving alternative pathways such as MAPK signaling [112]. A key aspect underlying these responses might be the well-established interplay between oxidative stress and ER stress, where each can amplify the other [113,114]. Notably, modulation of redox-sensitive components such as PDI with oxidants has been shown to alleviate ER stress [98], suggesting that oxidative stress response pathways are tightly integrated into ER stress responses and that redox regulation may play a compensatory role in contexts where canonical UPR signaling is not activated. Interestingly, HU has been shown to induce ER stress and trigger UPR activation in iPSC-derived dopaminergic neurons [115], and high doses of DIA also activate the UPR in *S. cerevisiae* [25]. However, our transcriptomic profile of DIA-treated *S. pombe* lacks evidence of UPR-related gene regulation, and this, together with the findings from Takano *and colleagues* and ours showing that HU does not induce UPR in yeast, suggests that the UPR is not universally engaged by thiol-oxidizing agents. These divergent responses may reflect inherent differences in UPR regulation across organisms [82,116].

We further demonstrate that, in addition to ER stress, HU and DIA treatments cause cytoplasmic proteotoxicity, as they lead to the formation of cytoplasmic foci containing chaperones and misfolding reporters. Pm, which blocks protein synthesis and releases nascent peptides from ribosomes, fully suppresses ER expansion but increases cytoplasmic protein aggregation, while CHX enhances ER expansion but fully prevents cytoplasmic protein aggregation, agreeing with previous studies showing that CHX impedes the formation of protein aggregates [71,117]. These observations suggest that nascent peptides attached to ribosomes are required for HU- or DIA-induced ER lumen expansion, and that cytoplasmic aggregates mostly arise from de novo protein synthesis during HU or DIA treatments. Cytoplasmic foci are not reverted by DTT addition (Fig 5F) nor suppressed by *gcs1* deletion (Fig 6G). HU treatment has been shown to result in the production of ROS including NO and H_2_O_2_ [43,44], which is produced in the cytoplasm in our experimental conditions (Fig 2D). HU also increased glutathionylation of cytoplasmic proteins, suggesting that the effect of HU on cytoplasmic proteostasis might involve different oxidative stressors and targets, whereas HU’s effect on ER proteostasis mainly impacts on redox protein folding through cysteine oxidation of luminal proteins, as has been recently shown [25]. Glutathionylation of proteins involved in translation, such as ribosomal proteins, and Hsp90 by HU could be relevant in the regulation of translation during stress, although the functional relevance of these modifications will require further investigations.

In summary, our data shows that HU causes thiol stress and disrupts redox homeostasis, which in turn impairs the folding of newly synthesized proteins. The molecular mechanisms by which HU achieves this phenotype require further investigation, but aberrant disulfide bond formation in ER luminal proteins and their defective retrotranslocation demonstrated by Takano *and colleagues* [25] likely contribute to ER expansion. All in all, our results uncover an unexpected off-target effect of HU unrelated to its primary target, RNR, which might be relevant in clinical pharmacology, as it could lead to side effects ranging from mild to life threatening, depending on patient-related risk factors.

## Methods

### Culture conditions

Standard methods for *S. pombe* growth and genetics were used [118]. Unless otherwise stated, liquid cultures were grown in YES medium at 30°C to mid-log phase in shaking incubators. Mating was performed on SPA solid medium plates, and the spores generated in the crosses were dissected using a dissection microscope (MSM 400; Singer Instruments) on YES agar plates.

### Strain construction

*S. pombe* strains used in this work are listed in Table 1. Combination of GFP-, mCherry- or tdTomato-tagged proteins and deletion mutants was generated by mating strains already available in our laboratory and posterior tetrad analyses. Gene tagging was performed using PCR- and homologous recombination-based methods as previously described [119]. Yeast transformation was performed by the lithium acetate method [120]. Unless otherwise stated, the proteins used and generated in this study were tagged at either the N-terminus or C-terminus and expressed from their endogenous loci. Constructs were checked by PCR. Fusion protein functionality was confirmed by testing strain growth on solid plates and liquid media. The strain expressing Sc CPY-GFP was generated as follows: the CPY ORF was amplified by PCR using Q5 DNA polymerase (New England Biolabs) and primers ScCPY_F (TTTCTCGAGATGAAAGCATTCACCAGTTTAC) and ScCPY_R (TTT GAA TTC TAA TAA GGA GAA ACC ACC GTG G) from *S. cerevisiae* genomic DNA. CPY* was amplified using the same primers from a plasmid containing S.c. CPY*-HA, kindly provided by Veit Goder. The PCR fragments were digested with EcoR1 and Xho1 and cloned in pAV0714 plasmid [119]. The new plasmids pUra^Afe1^-pact-CPY-sfGFP-term and pUra^Afe1^-pact-CPY*-sfGFP-term were linearized with Afe1 and transformed into wild-type U-cells. Positive integrants were selected in minimal media lacking uracil. To generate the strain expressing Pdi1-GFP, a fragment containing sfGFP-AHDL-bsdMX6 was amplified by PCR from pAV0773 [119], using Q5 DNA polymerase (NEB) and primers Pdi_sfGFP_0773_F (TCAAGAAGGAGAAAGAATCTGTTCCAGCACCCGATCTTGAGGATCAGGTCGCAGTGGAAGACGAAATGGCCGATGAGCTTATGAAGAAGTTCCAGCTATTTAGC) and Pdi_sfGFP_0773_R (TGTGTACTCATTTTATAAGGGACAGAAAAACAGAATGAAACTTGACAGACATGTAACTAAAGTTATAAAACTTTAAAGATCAGTATAGCGACCAGCATTCAC). This fragment was used to transform a wild type strain. Positive integrants were selected in media containing 15 µg/ml Blasticidin S (Labclinics. Cat. No. 14499).

**Table 1:** Strains used in this work.

Number	Genotype	Source
**RD257**	h- cdc25−22 cut11-GFP:ura	P. Nurse
**RD1936**	h+ wild-type U-L-	R. R. Daga
**RD4595**	h + pBip1-mCherry-AHDL::leu1 ura4-D18 ade6-	S. Oliferenko
**RD4596**	h- pBip1-mCherry-AHDL::leu1 ura4-D18 ade6-	S. Oliferenko
**RD6766**	h sid2-tomato-nat cut11-GFP-ura	R. R. Daga
**RD6911**	h + pBip1-mcherry-AHDL::leu1 cut11-GFP-ura	This work
**RD6912**	h- pBip1-mcherry- AHDL::leu1 cut11-GFP-ura	This work
**RD6990**	h cut11-GFP-ura nup211-mcherry-nat	R. R. Daga
**RD7174**	h pBip1-mCherry-ADHL::leu1 ish1-GFP-Kan	This work
**RD7255**	h pBip1-mcherry-AHDL::leu1 imp1-GFP-Kan	This work
**RD7555**	h heh1(lem2)-GFP-Kan pBip1-mcherry-ADHL::leu1	This work
**RD7733**	h + lem2::ura4 + ish1-GFP-KanMX6 pBip1-mcherry-ADHL::leu1	This work
**RD7735**	h kan-nmt41-GFP-ima1 pBip1-mcherry-ADHL::leu1	This work
**RD7737**	h man1-GFP-Hph pBip1-mcherry-ADHL::leu1	This work
**RD7924**	h nmt41-pap1-GFP-leu nup211-mCherry-Nat	This work
**RD7967**	h + cut11-GFP::ura4 + leu1–32	R. R. Daga
**RD7968**	h- cut11-GFP::ura4 + leu1–32	R. R. Daga
**RD7971**	h + hsp104-GFP-ura pBip1-mcherry-AHDL::leu1	This work
**RD7999**	h ssa1-YFP-Kan pBip1-mcherry-AHDL::leu1	This work
**RD8018**	h cdc22-M45 cut11-GFP-ura	NBRP
**RD8153**	h GFP-suc22 pBip1-mcherry-ADEL-leu	O. Nielsen
**RD8159**	h cdc22-CFP-Kan pBip1-mcherry-ADEL-leu	O. Nielsen
**RD8163**	h spd1::Hph cut11-GFP-ura	O. Nielsen
**RD8428**	[*S. cerevisiae*] nup84-mcherry rad52-GFP	R. Wellinger
**RD8436**	h gma12:GFP:ura pBip1:mcherry:ADEL:leu	R. R. Daga
**RD8723**	h nup120::nat cut11-GFP-ura pBip1-mcherry-ADHL::leu1	R. R. Daga
**RD8768**	h cmp7::kan cut11-GFP-ura pBip1-mCherry-AHDL::leu1	R. R. Daga
**RD8884**	h ire1::kan pBip1-mCherry-AHDL::leu1 cut11-GFP-ura	This work
**RD8971**	h vps4::Hph cut11-GFP-ura pBip1-mcherry-AHDL::leu1	R. R. Daga
**RD9060**	h- hsp16:GFP:NatMX6	E. Hidalgo
**RD9074**	h hsp16:GFP:NatMX6 pBip1-mcherry-AHDL::leu1	R. R. Daga
**RD9076**	h sty1::guk1–9-GFP::leu1 pBip1-mcherry-AHDL::leu1	E. Hidalgo
**RD9077**	h gcs1::KanMX6 cut11-GFP-ura pBip1-mcherry-AHDL::leu1	E. Hidalgo
**RD9102**	h sty1::rho1.C17R-GFP::leu1 alm1-tomato-nat	E. Hidalgo
**RD9135**	h gcs1::kan hsp104-GFP-ura alm1-tomato-nat	R. R. Daga
**RD9243**	h gsa1::kan cut11-GFP-ura pBip1-mCherry-AHDL::leu1	E. Hidalgo
**RD9281**	h bgs1::ura4 + Pbgs1::GFP-12A-bgs1:leu1 + pBip1-mCherry-ADEL-leu	J. C. Ribas
**RD9283**	h bgs4::ura4 + Pbgs4::GFP-12A-bgs4:leu1 + pBip1-mCherry-ADEL-leu	J. C. Ribas
**RD9285**	h ags1D 3’UTRags1::ags1-12A-GFP-12A:leu1 + :ura4 + pBip1-mCherry-ADEL-leu	J. C. Ribas
**RD9331**	h- bip1-GFP:leu leu- ura- his- ade-	H. Valdivieso
**RD9344**	h bip1-GFP:leu1 cut11-RFP-Hyg	This work
**RD9593**	h vgl1-GFP-Kan hsp104-RFP-Kan	This work
**RD9595**	h lys1 + :Pnmt41-GFP-atg8 pBip1-mCherry-AHDL-leu	This work
**RD9608**	h yop1-GFP-ura hsp104-mRFP:KanMX6	S. Oliferenko
**RD9610**	h ish1-mScarlet-HphMX6 hsp104:GFP:U	A. Fernández
**RD9612**	h sty1::rho1.C17R-GFP::leu1 hsp104-mRFP:KanMX6	E. Hidalgo
**RD9614**	h rtn1-GFP-ura4 hsp104-mRFP:KanMX6	S. Oliferenko
**RD9616**	h sty1::guk1–9-GFP::leu1 hsp104-mRFP:KanMX6	E. Hidalgo
**RD9618**	h gma12:GFP:ura hsp104-mRFP:KanMX6	This work
**RD9766**	h pdi1:sfGFP-AHDL-bsdMX A- L- U- (clon 5)	This work
**RD9795**	h (p)act1:CPY*:GFP-ura	This work
**RD9797**	h (p)act1:CPY:GFP-ura	This work

The strains used in this study were derived from original strains provided by F. Chang (University of California, San Francisco, CA), T. Toda (Graduate School of Integrated Sciences for Life, Hiroshima University, Higashi-Hiroshima, Hiroshima, Japan), J. Jiménez and V. Álvarez (Centro Andaluz de Biología del Desarrollo/Universidad Pablo de Olavide, Seville, Spain), P. Nurse (The Francis Crick Institute, London, UK), S. Moreno (Instituto de Biología Funcional y Genómica, Salamanca, Spain), Y. Watanabe (Institute of Molecular and Cellular Biosciences, University of Tokyo, Tokyo, Japan), I. Hagan (Cancer Research UK, University of Manchester, Manchester, UK), Y. Hiraoka (Advanced ICT Research Institute, Kobe, Japan; National Institute of Information and Communications Technology, Kobe, Japan/Graduate School of Frontier Biosciences, Osaka University, Suita, Japan), O. Niwa (Kazusa DNA Research Institute, Chiba, Japan), S. Oliferenko (The Francis Crick Institute, London, UK), J. C. Ribas (Instituto de Biología Funcional y Genómica, Salamanca, Spain), A. Fernández (Instituto de Biología Funcional y Genómica, Salamanca, Spain), R. Wellinger and H. Gaillard (Universidad de Sevilla, CABIMER, Sevilla, Spain) and the National Bio-Resource Project (Osaka, Japan).

### Cell synchronization

For synchronization in S phase using hydroxyurea (127-07-1; Sigma-Aldrich), cells were grown in YES media at 30 °C up to a OD_600_ of 0.3, and then HU was added from a 1M stock dissolved in YES liquid medium to a final concentration of 15 mM, keeping the culture at 30 °C for at least 3 hours. For *cdc25−22* thermosensitive strains (G2 arrest), cells were grown at 25 °C up to OD_600_ of 0.3 and then shifted to 36 °C for 4 hours.

### Viability assays

Viability assays, or drop assays, were performed by plating 5 μL of a OD_600_ = 0.3 cell culture of the indicated strain and then plating the same volume of three 1:10 sequential dilutions. Control plates consisted of YES agar and were incubated at 30 °C for 2–3 days. The rest of the plates were prepared by melting solid YES agar and adding the indicated drug to the concentration stated in the corresponding figure while the medium was still warm. After cooling, an exact replicate of the control disposition was plated and incubated at 30 °C for 2–3 days.

### Drug treatments

Hydroxyurea (127-07-1; Sigma-Aldrich) was used at a final concentration ranging from 15 to 200mM, as indicated in each experiment. Diamide (D3648; Sigma-Aldrich) was added to the sample at a final concentration of 3 mM. DETA-NONOate (A5581; Sigma-Aldrich) was added to the sample at a final concentration of 0.5 mM. Diethyl maleate (D97703; Sigma-Aldrich) was added to the sample at a final concentration of 0.5, 0.7 mM or 1 mM. Dithiothreitol (R0862; Thermo Scientific) was added to the sample at a final concentration of 1, 2, or 5 mM. Tunicamycin (T7765; Sigma-Aldrich) was added to the sample at a final concentration of 0.5 or 2 μg/mL. Cycloheximide (C7698; Sigma-Aldrich) was added to the sample at a final concentration of 100 μg/mL. Puromycin (BIP3340; Apollo Scientific) was added to the sample to a final concentration of 5 mM; cultures were pretreated for 30′ with this drug before adding DIA or HU to ensure complete inhibition of protein synthesis. L-Glutathione oxidized (G4501; Sigma-Aldrich) was added to the sample to a final concentration of 1 or 10 mM. Menadione (M5625; Sigma-Aldrich) was added to the sample to a final concentration of 50 μM. Hydrogen peroxide (H1009; Sigma-Aldrich) was added to the sample to a final concentration of 0.2, 1, or 5 mM. FM4-64 (T13320; Molecular Probes, Invitrogen) was added to a final concentration of 20 μM after a 3-hour 75 mM HU treatment, which was then incubated for an additional 15 min before drug washout.

### Protein extraction and western blot

For protein extraction, samples were prepared by trichloroacetic acid (TCA; Sigma; Cat. No. T0699) precipitation [121]. In brief, 50 mL cultures were harvested at exponential phase by centrifugation, then cells were incubated in cold 10% TCA for at least 30 min, after which they were washed with 100% cold acetone and dried. The dried cell pellet was then resuspended and lysed in beating buffer (8 M urea, 50 mM ammonium bicarbonate, 5 mM EDTA) with chilled acid-washed glass beads (Sigma; Cat. No. G8772) in a FastPrep-24 (MP Biomedicals) at 4 °C four times during 30 seconds. Lysates were cleared by centrifugation at 4 °C and proteins in the supernatant were quantified using the Pierce BCA Protein Assay Kit (Sigma; Cat. No. 23225). Protein samples were adjusted to the same total protein concentration (30 micrograms for Bip1-GFP, 50 micrograms for the rest of the samples), resuspended in 2X-SDS-Sample buffer + DTT and boiled. Protein electrophoresis was performed using 10% TGX-Fast Cast acrylamide gels (Bio-Rad; Cat. No. 161-0183) and blotted on nitrocellulose membranes (Bio-Rad; Trans-Blot Turbo Transfer System). Membranes were blocked with TBS + 0.1% Tween 20 (T-TBS) + 5% (w/v) non-fat dry milk, then probed overnight with an anti-GFP primary antibody (Anti-GFP; mouse monoclonal primary antibody; Roche 11,814,460,001) diluted 1:1000 in 5% non-fat milk. After three washes using T-TBS, membranes were incubated with the corresponding secondary antibody (Anti-mouse IgG-HRP; Sigma A3562) diluted 1:2000 in 5% non-fat milk. Stain-free staining was used as loading control, while protein detection was performed using Supersignal West Femto (Thermo Fisher; Cat. No. 34095) and a Chemidoc XRS+ (Bio-Rad).

### RNA-Seq and transcriptomics analysis

RNA was extracted from 200 mL cultures grown in agitation at 30 °C, then incubated under the desired conditions (untreated cultures [C-]; cultures treated with 75 mM HU for 60 min [HU60] and for 150 min [HU150]; and cultures treated with 3 mM DIA for 30 min [DIA30] and for 60 min [DIA60]). HU60 and DIA30 correspond to a time prior N-Cap formation; HU150 and DIA60 mark a time point at which cells begin to show the phenotype). Cells were then pelleted and ruptured using a precooled mortar with liquid nitrogen. After cell lysis, debris was recovered and snap-frozen in liquid nitrogen. RNAase-free water was then added to the pellets and RNA was extracted using the RNAeasy kit (74,104; Qiagen). Final RNA concentration was adjusted to 5 ng and sent to BGI for sequencing. The bulk data were then processed using a customized BaSH script. Read assembly was performed using STAR 2.7.10a [122], mapping was performed on the *S. pombe* genome version ASM294v2.48 using SAMtools 1.3 [123], and the final count matrix was performed with htseq-count from package HTSeq 2.0.1 [124]. For the search of differentially expressed genes, a script in the R programming language was used, using Bioconductor 3.19 and the DESeq2 package [125]. A gene was considered as differentially expressed when it showed an absolute value of log2FC(LFC)≥1 and an adjusted p-value<0.05, the p-value being corrected for multiple testing using the Benjamini-Hochberg method. P-values were automatically modulated by default with DESeq2. The GO term enrichment was performed using DAVID [126] and ShinyGO [127]. Raw data from the RNA-Seq analysis is publicly available in *Gene Expression Omnibus* under the accession number GSE309439.

### *In vivo* measurement of H_2_O_2_ levels with redox biosensors

Auxotrophic strain HM123 (*h*^*−*^ *leu1–32*, lab stock) was transformed with each specific plasmid and grown in filtered minimal media at 30 °C as previously described [45]. Stationary phase pre-cultures were diluted to reach an OD_600_ of 1 after 4–5 duplications. We determined the degree of sensor oxidation (OxD), as previously described [128] being 1 the maximum oxidation of the probe reached with 1 mM H_2_O_2_ treatment, and 0 the complete reduction of the biosensor with 50 mM DTT. After recording 4 cycles, treatments were added as follows (final concentrations): water as control, 0.1 mM H_2_O_2_, 3 mM of DIA, and 75 mM HU.

### Microscopy and image analysis

Live cell imaging was performed at 25°C, with a Roper Scientific spinning disk confocal microscope (IX-81; Olympus; CoolSnap HQ2 camera, Plan Apochromat 100 × , 1.4 NA objective), with Metamorph software (Molecular Devices), or with a spinning disk 3i (Intelligent Imaging Innovations) (Zeiss Axio Observer 7 inverted, PRIME 95B camera, 1.46 NA objective; Zeiss) with SlideBook6 software. Images were analyzed with ImageJ/Fiji (National Institutes of Health). For imaging, cells were immobilized in soybean lectin (Sigma)-coated μ-Slide 8-well dishes (Ibidi; Cat. No. 80827) in the case of *S. pombe*; immobilization *of S. cerevisiae* was achieved by placing a YPD + agar 1% pad upon the cells in the μ-Slide 8-well dishes. Unless otherwise stated, images shown are Z projections of 3 central z sections with a step size of 0.3 μm. Quantifications of total fluorescence intensity were performed on SUM projections of 18 z sections with a step size of 0.3 µm.

Roundness measurements were performed in a single central z-section. The “roundness” parameter in the ‘Shape Descriptors’ plugin of Fiji/ImageJ was used after applying a threshold to the image in order to select only the more intense regions and subtract background noise [129]. Roundness descriptor follows the function:


$$Round=\text{4}\times \frac{\left[Area\right]}{\pi \times [Major\;axis{]}^{\text{2}}}$$

where [*Area*] constitutes the area of an ellipse fitted to the selected region in the image and [*Major axis*] is the axis that connects the two vertexes that are farther apart in that same ellipse.

To quantify the aggregated area, as tagged with Hsp104-GFP, in cells treated with DIA or HU, Pm or a combination of Pm with each of the drugs, a SUM projection of 18 Z slices was performed, the total area of the cells was measured (in px^2^), and then a threshold was applied to select the areas of highest intensity, manually adjusting the parameters to select the aggregates. The area of these aggregates was also measured (px^2^) and divided by the total cell area to obtain the percentage of the cellular area that corresponds to Hsp104-GFP aggregates.

FRAP experiments in S1A Fig were performed by bleaching the whole nucleus and measuring how cytoplasmic Imp1-GFP fluorescence intensity progressed over time. FRAP experiments in S1B Fig were performed by bleaching the whole cytoplasm (to the extent possible) and measuring how Imp1-GFP fluorescence intensity progressed over time in the nucleoplasm. FRAP experiments in S4A Fig were performed by bleaching the whole nucleus and measuring perinuclear Bip1 fluorescence intensity progression over time, for which we selected the periplasmic region (either isometric or expanded) with a threshold. FRAP experiments in S4B Fig were performed by selecting half of a nucleus with expanded ER and bleaching that half, and then measuring how Bip1 fluorescence intensity progressed over time in each of the two halves (bleached and unbleached) by manually selecting each one.

### Transmission electron microscopy

This protocol, based on previous works [130,131], was performed using the Leica EM AFS2 device for freeze substitution resin-embedding at low temperatures. *S. pombe* samples were subjected to high-pressure freezing using the Leica EM HPM100 system, and then transferred from liquid nitrogen to the AFS2 chamber, where the freeze substitution was initiated. Samples were washed thrice with cold acetone and post-fixed with 1% osmium tetroxide (dissolved in acetone) for 2 hours at 4 °C in darkness, after which they were again washed thrice with acetone. Spurr resin (prepared as described by Spurr [132]) inclusion was then initiated as follows: 1:1 resin/acetone overnight at room temperature, 3:1 resin/acetone 4–8 hours at room temperature, 100% resin overnight at room temperature. After that, samples are plated with 100% resin and polymerized for 2–3 days (at least 24 hours) at 70 °C. Electron microscopy images were then acquired.

### HT1080 fibrosarcoma cell preparation and cultivation

HT1080 fibrosarcoma cells were cultured in DMEM medium supplemented with 10% fetal bovine serum (FBS; Capricorn Scientific, Ebsdorfergrund, Germany), a combination of 10,000 IU/mL penicillin and 10,000 μg/mL streptomycin (Corning, MA, USA), and 2 mM L-glutamine (Merck, Darmstadt, Germany). The cells were maintained at 37°C in a 5% CO_2_ humidified atmosphere. Cultures were grown in flasks until they reached 70%–80% confluence. Subsequently, 8,500 cells were seeded per well in a 96-well plate with a final volume of 200 μL and incubated overnight prior to conducting various endoplasmic reticulum assays.

### High content screening analysis of endoplasmic reticulum stress

Images were captured using the Operetta High Content Imaging System (PerkinElmer). Images were collected at different time points (0, 12, and 24 hours) from various wells to assess the duration of cell exposure to the compounds. Fluorescent dyes (Hoechst 33342 [H1399; Invitrogen, Thermo Fisher Scientific], for DNA, added at a working concentration of 2.5 μM; CellMask Deep Red [C10046; Invitrogen, Thermo Fisher Scientific], for the plasma membrane, added at a working concentration of 0.25 μM; ER-ID Red assay kit [ENZ-51025-K500; Enzo Life Sciences], for the endoplasmic reticulum, added at a working concentration of 1:2000) were uniformly added and incubated for 1 hour before imaging. Image analysis was conducted using Harmony software version 4.8 (PerkinElmer). Cells that moved out of the selected field of view or underwent division, either during initial imaging or at later time points, were excluded from the analysis. The signal was corrected by subtracting the cytoplasmic signal ($S_{cyto}$) from the ER signal ($S_{ER}$), and the external signal around each cell ($S_{surround})$ was then subtracted from this adjusted ER signal. This process ensures that the final intensity measurement ($S_{corrected}$) accurately reflects the corrected ER signal isolated from both the cytoplasmic and surrounding signals:


$$S_{corrected}=\left(S_{ER}-S_{cyto}\right)-S_{surround}\;$$

Each well was divided into 4–5 distinct fields of view to ensure comprehensive data collection. The acquired images from the Operetta High Content Imaging System were analyzed and processed using FIJI/ImageJ.

### Quantification and statistical analysis of microscopy images

At least three independent biological replicates were performed for each experiment. Graphs and statistical analyzes were performed with GraphPad Prism 5.0 (GraphPad Software) and Excel (Microsoft). Unless otherwise stated, graphs represent the mean and error bars represent the standard deviation (SD). n is the total number of cells scored in each repetition of the experiment, as described in each figure legend. Statistical comparison between groups in graphs depicting N-Cap incidence and violin plots was performed by unpaired Student *t* test (for normal distributions) or Mann-Whitney’s U-test (for non-Gaussian distributions), considering two-tailed p-values exceeding 0.05 to be not significant. Statistical comparison between groups in graphs accounting for number of foci was performed by chi-squared test. Asterisks in graphs correspond to the following *p*-values: (ns) *P* > 0.05; (*) *P* ≤ 0.05; (**) *P* ≤ 0.01; (***) *P* ≤ 0.001; (****) *P* ≤ 0.0001.

### Detection of glutathionylated proteins by mass spectrometry

We are thankful to the Research Support Central Services (SCAI) of the University of Málaga (Spain) for providing the necessary infrastructural support for the successful accomplishment of this research work.

#### Protein extraction.

For protein extraction for mass spectrometry analysis of glutathionylation, 50 mL of each culture was centrifuged at room temperature, and the pellets were washed with 1 mL of 1 × phosphate-buffered saline (PBS). The washed pellets were then transferred to a screw-cap microcentrifuge tube and centrifuged for 3 min at 4,000 rpm. After discarding the supernatants, pellets were immediately frozen in liquid nitrogen. The frozen pellets were thawed on ice and resuspended in 150 µL of RIPA buffer (50 mM Tris-HCl, pH 8.0, 150 mM NaCl, 0,1% SDS, 0,5% sodium deoxycholate, and 1% NP-40). Cold glass beads were then added, and cells were disrupted using a FastPrep with two pulses of 45 seconds, keeping the samples on ice between pulses. The lysates were eluted into cold 2 mL microcentrifuge tubes and centrifuged at 13,000 rpm for 5 min at 4°C, and the supernatants were collected for further analysis.

#### Gel-assisted digestion and peptide extraction.

Gel-assisted proteolysis was performed by trapping the protein solution in a polyacrylamide gel matrix. For this, 45 µL of sample (1 µg/µL) was mixed with 14 µL of 40% acrylamide monomer solution, followed by the rapid addition of 2.5 µL of 10% ammonium persulfate and 1 µL of TEMED. The mixture was allowed to fully polymerize for 20 min before performing in-gel digestion. Using a scalpel, the gels were cut into 1–2 mm cubes and treated with 50% acetonitrile (ACN)/ 25 mM ammonium bicarbonate. The samples were then dehydrated, dried with ACN, and cysteine residues were carbamidomethylated with 55 mM iodoacetamide in 50 mM ammonium bicarbonate for 20 min at room temperature in the dark. After carbamidomethylation, the gel pieces were dehydrated again, and proteins were digested by rehydrating the gel pieces in a 10 ng/µL trypsin solution (Promega) and incubating them at 30°C overnight. Peptides were extracted from the gel pieces with 0.1% formic acid (FA) in ACN for 30 min at room temperature. The samples were dried in a SpeedVac vacuum concentrator to remove residual ACN and ammonium bicarbonate, redissolved in 0.1% FA, and sonicated for 3 min. They were then centrifuged at 13,000 *g* for 5 min. Finally, the samples were purified and concentrated using C18 ZipTip (Merck) according to the manufacturer’s instructions before being transferred to the injection vial.

#### Liquid chromatography and mass spectrometry.

The samples were injected into an *Easy nLC 1200 ultra-high-performance liquid chromatography (UHPLC)* system coupled to a *Q-Exactive HF-X* quadrupole-linear ion trap-Orbitrap hybrid mass spectrometer (ThermoFisher Scientific). The software versions used for data acquisition and operation were *Tune 2.9* and *Xcalibur 4.1.31.9*. The UHPLC mobile phases were as follows: solvent A consisted of 0.1% FA in water, and solvent B consisted of 0.1% FA in 80% ACN. From a temperature-controlled autosampler, 1 µL (100 ng) of the peptide mixture was automatically loaded onto a precolumn (*Acclaim PepMap 100*, 75 µm × 2 cm, C18, 3 µm, 100 Å, ThermoFisher Scientific) at a flow rate of 20 µL/min and eluted along a 50 cm analytical column (*PepMap RSLC* C18, 2 µm, 100 Å, 75 µm × 50 cm, ThermoFisher Scientific). Peptides were eluted from the analytical column using a 180-minute gradient from 5% to 20% solvent B, followed by a 5-minute gradient from 20% to 32% solvent B, and finally up to 95% solvent B for 10 min before re-equilibrating with 5% solvent B at a constant flow rate of 300 nL/min. The *LTQ Velos ESI Positive Ion Calibration Solution* (Pierce, IL, USA) was used for external calibration of the instrument before sample analysis, and an internal calibration was performed using the polysiloxane ion signal at m/z 445.120024 from ambient air. MS^1^ scans were performed in the m/z range of 375–1,600 at a resolution of 120,000. Using a data-dependent acquisition mode, the 15 most intense precursor ions with a charge of +2 to +5 were isolated within a 1.2 m/z window and fragmented to obtain the corresponding MS^2^ spectra. Fragment ions were generated in a high-energy collision dissociation (HCD) cell with a first fixed mass at 110 m/z and detected in an Orbitrap mass analyzer at a resolution of 30,000. Dynamic exclusion for selected ions was set to 30 s. The maximum ion accumulation time allowed in MS and MS2 mode was 50 ms and 70 ms, respectively. Automatic gain control was used to prevent ion trap overfilling and was set at 3 × 10^6^ ions and 2 × 10^5^ ions for a full MS and MS^2^ scans, respectively.

#### Protein identification and label-free quantification.

MS/MS2 spectra searches were performed against the *S. pombe 972h UniProtKB database, including isoforms (version 2025-02-05)*. Raw data were analyzed using *Proteome Discoverer 2.5* (Thermo Fisher Scientific) with the Sequest HT search engine, using mass tolerances of 10 ppm and 0.02 Da for precursor and fragment ions, respectively. Up to two missed tryptic cleavage sites were allowed. Variable modifications included methionine oxidation, N-terminal acetylation, and carbamidomethylation, glutathionylation, and nitrosylation of cysteine residues; no fixed modifications were set. The false discovery rate (FDR) for peptide and protein assignments was determined using the Percolator software package (included in *Proteome Discoverer 2.5*), based on a target-decoy approach using an inverted protein database as a decoy, with a strict FDR threshold of 1%. Results were filtered to accept only proteins with at least two unique peptide sequences.

Label-free quantification was implemented using the *Minora* feature in *Proteome Discoverer 2.5*, with the following parameters: a maximum retention time alignment of 10 min and a minimum signal-to-noise ratio of 5 for feature linking. Abundances were based on precursor ion intensities. For total protein quantification, sample normalization was performed based on the total abundance of peptides and proteins, while for modification quantification, the total abundance of modified peptides was used. The normalized abundance values of each protein were scaled to 100. The relative normalized and scaled abundance of each protein was expressed as the mean ± standard deviation (SD) of biological replicates. Protein abundance ratios were calculated based on unique peptides, using the mean of abundances in each condition. *p*-values for abundance ratios were calculated using an ANOVA based on individual proteins.

## Supporting information

S1 Data Quantitative observations that underlie the data summarized in the figures. XLXS file containing raw data of all replicates for each figure and supplementary figure appearing in-text. The file is divided into sheets named after the corresponding figure or panel. In the file, SD stands for standard deviation, which is used as a measurement for the statistical error. Statistical comparison between groups in graphs depicting N-Cap incidence and violin plots was performed by unpaired Student *t* test (for normal distributions) or Mann-Whitney’s u test (for non-Gaussian distributions), considering two-tailed p-values exceeding 0.05 to be not significant. Statistical comparison between groups in graphs accounting for number of foci was performed by chi-squared test.
(XLSX)

S1 Fig NPCs clustered after HU treatment remain functional for nucleocytoplasmic transport and their formation is independent of S phase arrest induced by RNR inhibition. **(A)** Recovery of importin (Imp1-GFP) fluorescence after photobleaching in the nucleus (left) and in the cytoplasm (right) in cells with and without clustered NPCs exposed to 15 mM HU for 4 hours. Graphs represent the mean ± SD of the fluorescence intensity of Imp1-GFP, normalized to the fluorescence in the indicated compartment right before bleaching, and measured in at least 10 cells of each phenotype. Orange dotted areas mean the placing of the region in which fluorescence intensity is measured. Black dotted areas mean the bleached areas. **(B)** Progression of GFP-Pap1 intensity after exposure and removal of 0.2 mM H_2_O_2_, in cells with and without clustered NPCs, after a 4-hour exposure to 15 mM HU. Graph represents the mean ± SD of the fluorescence intensity of GFP-Pap1, normalized to the background and measured in at least 15 cells of each phenotype. **(C)** Representative confocal microscopy image of a *spd1Δ* mutant strain after 4 hours in 75 mM HU showing clustered NPCs (left), and graph comparing NPC cluster formation in a wild-type strain and a *spd1Δ* mutant (right). Images are SUM projections of three central Z slices. Scale bars represent 5 microns. The graph shows the mean ± SD of two independent repetitions of the experiment, and in each repetition at least 100 cells were accounted for each condition. **(D) Left:** Representative confocal microscopy image of a *cdc22-M45* thermosensitive mutant after 4 hours at restrictive temperature (36 °C) proving that these cells do not form NPC clusters per se (left), and graph comparing NPC cluster formation in a control wild-type strain exposed to 75 mM HU at 37 °C and a *cdc22-M45* mutant kept at 36 °C for 4 hours and then exposed to 75 mM HU for the following 3 hours while still at restrictive temperature (right). Images are SUM projections of three central Z slices. Scale bars represent 5 microns. Graph shows the mean ± SD of two independent repetitions of the experiment, and in each repetition at least 100 cells were accounted for each condition. **(E)** Representative confocal microscopy images of a wild-type and a *cdc25−22* thermosensitive mutant, both expressing Cut11-GFP, at restrictive temperature (36 °C) after incubation for 4 hours (left) to achieve full blockage of the mutant in the G2/M transition, and after addition of HU for 3 extra hours while kept at 36 °C so cell cycle in the mutant remains blocked (right). When *cdc25−22* cells are blocked in G2/M transition by temperature shift and treated with HU during this blockage, cells form NPC clusters while in G2/M. Magenta asterisks indicate cells with NPC clustering. Images are SUM projections of five central Z slices. Scale bars represent 5 microns. Source data for this figure can be found in S1 Data.
(PDF)

S2 Fig N-Cap dispersion requires perinuclear ER redistribution, and ER expansion causes INM and ONM/ER membrane separation. **(A)** Quantification of N-Cap reposition time after HU washout following a 4-hour incubation in 75 mM HU, comparing cells with a more expanded ER (‘large ER’; mean area = 4.5 ± 0.4 μm^2^) versus cells with a less expanded ER (‘small ER’; mean area = 2.5 ± 0.6 μm^2^). Cells were considered to have a ‘large ER’ when their ER area along the projection of a 3-Z section was over 4 μm^2^. Graph shows violin plots with the mean ± SD of the reposition time of N-Caps of at least 20 cells with each of the forementioned perinuclear ER morphologies. **(B)** Timelapse following a group of cells tagged with mCherry-AHDL, previously exposed to 75 mM HU for 4 hours, after drug washout. Cells with a larger ER take longer to recover the even perinuclear architecture than those with a less-expanded ER. Asterisks mark when a cell recovers its normal perinuclear architecture. Images are SUM projections of three central Z slices. Scale bars represent 5 microns. **(C-D)** Confocal microscopy images of cells expressing INM tags Lem2-GFP and GFP-Ima1 (C), and Man1-GFP (E), opposed to the luminal ER marker mCherry-AHDL, after a 4-hour incubation in 75 mM HU. Magenta arrows show the presence of membranes surrounding the ER lumen. Images are SUM projections of three central Z slices. Scale bars represent 5 microns. **(E-F)** Transmission electron images (TEM) of a wild-type strain in control conditions (E) and after a 4-hour incubation in 75 mM HU (F). Insets focus on nuclei in each condition; magenta arrows indicate inner nuclear membranes (INM) and outer nuclear membranes continuous with the ER (ONM/ER). N = nucleus; CW, cell wall; NE, nuclear envelope; Cyt. = cytosol. Scale bars represent 1 micrometer, 500 nm or 100 nm, as indicated in each panel. **(G)** Images of cells expressing mCherry-AHDL and either Cut11-GFP or Ish1-GFP, comparing a wild-type strain with N-Caps to mutant strains *cmp7Δ*, *lem2Δ* or *vps4Δ*, all of which show a phenotype of fragmented ER, after treatment with 75 mM HU for 3 hours. **(H)** Quantification of the incidence of N-Caps and fragmented ER phenotypes in the total population of cells in the previously addressed conditions. Graphs show the mean ± SD of at least 50 cells in two independent repetitions of the experiment. Source data for this figure can be found in S1 Data.
(PDF)

S3 Fig HU-induced perinuclear architecture alterations are evolutionarily conserved. **(A)** Comparison of *S. cerevisiae* cells expressing mCherry-tagged NUP84 in control conditions (left) and after 3 hours in HU 100mM (right). The asterisk marks a cell with clustered NPCs. Confocal microscopy images are SUM projections of three central Z slices. Scale bar represents 5 µm. **(B)** In a *S. cerevisiae* nucleus tagged with NUP84-mCherry, NPCs eventually recover their even distribution along the NE following drug washout after a 3-hour incubation in 100 mM HU. t = time after HU washout. Confocal microscopy images are SUM projections of three central Z slices. Scale bar represents 5 µm. **(C) Upper panel:** Merge of fluorescence images showing Hoescht 333,248 (blue, nucleus) and ER-ID Red (yellow, endoplasmic reticulum) at 0, 12 and 24 hours post-treatment with HU at 200 µM and 1 mM, DTT 1 mM or the equivalent diluent (control). Scale bars represent 100 µm. **Lower panel:** Cells were monitored at 0, 12, and 24 hours post Hydroxyurea (HU) treatment, with 3 wells and 5 fields per well counted per time condition. Dyes were applied 45 min before each measurement. ER intensity was segmented to correct for cytoplasmic and extracellular signals, and normalized by ER area in square micrometers (µm²), represented as ‘Total intensity’ (AU × µm²). The control condition (C) corresponds to the highest concentration of HU diluent (water). The number of cells analyzed per experimental condition: 0h: C (959), DTT (1,333), HU 200 µM (1,577), HU 1 mM (1,461). **(D) Upper panel:** Merge of fluorescence images showing Hoescht 333,248 (blue, nucleus) and ER-ID Red (yellow, ER) at 60 min, 90 min, and 120 min post-treatment with DIA at 50 µM, DTT 100 µM or the equivalent diluent (control), representing the same cells over time. Scale bar represents 100 µm. **Lower panel:** Cells were monitored from 60 to 120 min after compound and dye addition, segmented to measure ER intensity corrected for cytoplasmic and extracellular surrounding signals and normalized by ER area (in µm²), represented as ‘Total intensity’ (AU × µm²). The number of cells analyzed per experimental condition was derived from three independent wells: DIA: Treatment with Diamide (1,335 cells). Control: Control group receiving equivalent concentrations of diluents (1,792 cells). DTT: Treatment with DTT (1377 cells). Source data for this figure can be found in S1 Data.
(PDF)

S4 Fig Bip1-GFP mobility is reduced during HU or DIA treatments, and cytoplasmic foci are not membrane-bound nor correspond to canonical heat shock induced stress granules. **(A)** FRAP experiment consisting of photobleaching of the perinuclear ER to measure Bip1-GFP fluorescence recovery either in untreated conditions, in 3 mM DIA after a 4-hour incubation, or in 75 mM HU also after a 4-hour incubation. Cells with expanded ER (N-Cap) showed almost no fluorescence recovery in comparison with cells without N-Cap and in control conditions. The graph represents the mean ± SD of the fluorescence intensity of Imp1-GFP, normalized to the fluorescence in the indicated compartment right before bleaching, and measured in at least 10 cells of each phenotype. **(B)** FRAP experiment consisting of photobleaching half of the perinuclear ER to measure Bip1-GFP fluorescence recovery in both sides of the compartment. The graph represents the mean ± SD of the fluorescence intensity of Imp1-GFP, normalized to the fluorescence in the indicated compartment right before bleaching, and measured in at least 10 cells of each phenotype. **(C)** Confocal microscopy images of cells expressing Vgl1-GFP and Hsp104-mRFP in untreated conditions and after 4 hours in 75 mM HU or 3 mM DIA. Insets below show individual Hsp104-mRFP aggregates and show that Vgl1-GFP is not part of the aggregate. **(D)** Images of cells expressing Hsp104-GFP and Ish1-mScarlet in untreated conditions and after 4 hours in 75 mM HU or 3 mM DIA. Insets below show that Hsp104 aggregates are not surrounded by Ish1-containing membrane. **(E-F)** Images of cells expressing Hsp104-mRFP and either Rtn1-GFP (E) or Yop1-GFP (F) in untreated conditions and after 4 hours in either 75 mM HU or 3 mM DIA. Insets below show that Hsp104-containing aggregates are not surrounded by transmembrane ER proteins. **(G)** Images of cells expressing Gma12-GFP and Hsp104-mRFP in untreated conditions and after 4 hours in either 75 mM HU or 3 mM DIA. Insets on the right show that Hsp104-mRFP aggregates do not colocalize with Gma12-GFP foci. Confocal microscopy images are SUM projections of 3 central Z slices. Scale bar represents 5 μm. Source data for this figure can be found in S1 Data.
(PDF)

S5 Fig Anisomycin recapitulates CHX behavior when combined with HU or DIA. **(A)** Quantification of the incidence of the N-Cap phenotype in a wild-type strain during 3 mM DIA treatment (upper graph) or 75 mM HU treatment (lower graph) when combined with 100 μg/mL anisomycin (ANM). Graphs show the mean ± SD of two independent repetitions of the experiment, and in each repetition at least 100 cells were accounted for each condition. **(B)** Confocal microscopy images of cells expressing Hsp104-GFP (green) and mCherry-AHDL (magenta) after a 3-hour incubation in either control conditions, 100 μg/mL ANM, 3 mM DIA, 75 mM HU, DIA and ANM, or HU and ANM. Hsp104-GFP aggregates are detected only in DIA and HU treatments. Images are SUM projections of 3 central Z slices. Scale bars represent 5 μm. Source data for this figure can be found in S1 Data.
(PDF)

S6 Fig HU and DIA induce a UPR-independent response. **(A)** Canonical ER stress as seen with the luminal ER tag mCherry-AHDL in a wild-type strain after treatment with DTT 2 mM and 5 mM, and with tunicamycin (Tn) 0.6 μg/mL and 2 μg/mL, after a 2-hour incubation. Images are SUM projections of three central Z slices. Scale bars represent 5 microns. **(B)** Upper panels: Confocal microscopy images of a representative cell of a wild-type strain and an *ire1Δ* strain, both expressing Cut11-GFP and mCherry-AHDL and showing N-Caps after incubation in 3 mM DIA for 4 hours. Images are SUM projections of three central Z slices. Scale bars represent 5 microns. Lower panels: Quantification of the incidence of the N-Cap phenotype in a wild-type strain and an *ire1Δ* strain during 3 mM DIA treatment. Graph shows the mean ± SD of three independent repetitions of the experiment, and in each repetition at least 100 cells were accounted for each condition. **(C)** Viability assay of a wild-type strain and an *ire1Δ* strain in control conditions, in presence of 2.5 mM DTT and in 3 mM DIA after a 2-day incubation at 30 °C. **(D)** Principal component analysis separating the three independent repetitions of each of the five conditions addressed in the RNA-Seq. Replicates are very consistent, and HU and DIA conditions are well-differentiated between them and with respect to the control. **(E)** Heatmap with the expression profile of upregulated and downregulated genes in 3 mM DIA after 30 and 60 min, and in 75 mM HU after 60 and 150 min, with respect to an untreated culture. Three replicates of each condition were assessed. Blue indicates upregulation; yellow indicates downregulation; LFC = Log2 fold change. **(F)** Volcano plots detailing the significantly upregulated and downregulated genes (LFC ≥ 1 or LFC ≤ −1 respectively, p-value<0.05) in DIA (upper graph) and HU (lower graph), pooling together both times addressed. Red dots show genes that surpass the set thresholds of LFC and p-value. Highlighted genes are some of the common genes in all conditions; ‘variables’ stands for the total number of genes. **(G)** Upset plots for comparing genes that are upregulated (upper graph) and downregulated (lower graph) between the four treatment conditions. The numbers indicate the genes that are common to the designated combination of conditions. In the case of the unconnected dots, numbers show genes exclusive to that condition alone. Set size graphs indicate the total number of genes in each condition. **(H)** GO term enrichment for the biological processes shared among the genes common to the four treatment conditions. **(I)** GO term enrichment for the molecular functions shared among the genes common to the four treatment conditions. Source data for this figure can be found in S1 Data. Raw data from the RNA-Seq analysis is publicly available in *Gene Expression Omnibus* under the accession number GSE309439.
(PDF)

S1 Table HU and DIA induce protein glutathionylation. Quantification of the total amount of proteins and peptide groups detected in each of the three experimental conditions (C-: untreated control; DIA: 3-hour incubation in 3 mM DIA; HU: 3-hour incubation in 75 mM HU).
(PDF)

S2 Table Proteins glutathionylated in HU. Summary of the proteins detected as glutathionylated by mass spectrometry after a 3-hour treatment with 75 mM HU. Sum PEP Score represents the protein score, calculated as the sum of the negative logarithm of the PEP values of the connected PSMs. The PEP (Posterior Error Probability) indicates the probability that a reported match is a random event (higher values indicate greater confidence in identification). Coverage (%) refers to the sequence coverage percentage, calculated by dividing the number of amino acids in all identified peptides by the total number of amino acids in the full protein sequence (higher values indicate better coverage).
(PDF)

S3 Table Proteins glutathionylated in DIA. Summary of the proteins detected as glutathionylated by mass spectrometry after a 3-hour treatment with 3 mM DIA. Sum PEP Score represents the protein score, calculated as the sum of the negative logarithm of the PEP values of the connected PSMs. The PEP (Posterior Error Probability) indicates the probability that a reported match is a random event (higher values indicate greater confidence in identification). Coverage (%) refers to the sequence coverage percentage, calculated by dividing the number of amino acids in all identified peptides by the total number of amino acids in the full protein sequence (higher values indicate better coverage).
(PDF)

S4 Table Proteins glutathionylated in untreated conditions. Summary of the proteins detected as glutathionylated by mass spectrometry in an untreated culture. Sum PEP Score represents the protein score, calculated as the sum of the negative logarithm of the PEP values of the connected PSMs. The PEP (Posterior Error Probability) indicates the probability that a reported match is a random event (higher values indicate greater confidence in identification). Coverage (%) refers to the sequence coverage percentage, calculated by dividing the number of amino acids in all identified peptides by the total number of amino acids in the full protein sequence (higher values indicate better coverage).
(PDF)

S5 Table Summary of proteins glutathionylated both in HU and DIA. Proteins common to both HU and DIA treatment, with their detailed cellular localization and involvement in biological pathways.
(PDF)

S1 Raw Images Uncropped western blot images included in Fig 4B, 4D, 4E and 4F. (PDF)

## Abbreviation:

DIA: diamide
ERAD: ER-associated degradation
FRAP: fluorescence recovery after photobleaching
GSH: Glutathione
HSPs: heat shock proteins
HU: Hydroxyurea
INM: inner nuclear membrane
MMP2: matrix metallopeptidase 2
MD: menadione
NE: nuclear envelope
NPCs: nuclear pore complexes
ONM: outer nuclear membrane
GSSG: oxidized glutathione
ROS: reactive oxygen species
RNR: ribonucleotide reductase
SPB: spindle pole body
TEM: transmission electron microscopy
UPR: Unfolded Protein Response.
