# Supplementary material for: Hydroxyurea induces an oxidative stress response that triggers ER expansion and cytoplasmic protein aggregation
Source: PLoS Biol. 2025 Nov 19;23(11):e3003493. doi: 10.1371/journal.pbio.3003493 (PMC12654915; doi:10.1371/journal.pbio.3003493)
Supplement: S1 Fig — (A) Recovery of importin (Imp1-GFP) fluorescence after photobleaching in the nucleus (left) and in the cytoplasm (right) in cells with and without clustered NPCs exposed to 15 mM HU for 4 hours. Graphs represent the mean ± SD of the fluorescence intensity of Imp1-GFP, normalized to the fluorescence in the indicated compartment right before bleaching, and measured in at least 10 cells of each phenotype. Orange dotted areas mean the placing of the region in which fluorescence intensity is measured. Black dotted areas mean the bleached areas. (B) Progression of GFP-Pap1 intensity after exposure and removal of 0.2 mM H2O2, in cells with and without clustered NPCs, after a 4-hour exposure to 15 mM HU. Graph represents the mean ± SD of the fluorescence intensity of GFP-Pap1, normalized to the background and measured in at least 15 cells of each phenotype. (C) Representative confocal microscopy image of a spd1Δ mutant strain after 4 hours in 75 mM HU showing clustered NPCs (left), and graph comparing NPC cluster formation in a wild-type strain and a spd1Δ mutant (right). Images are SUM projections of three central Z slices. Scale bars represent 5 microns. The graph shows the mean ± SD of two independent repetitions of the experiment, and in each repetition at least 100 cells were accounted for each condition. (D) Left: Representative confocal microscopy image of a cdc22-M45 thermosensitive mutant after 4 hours at restrictive temperature (36 °C) proving that these cells do not form NPC clusters per se (left), and graph comparing NPC cluster formation in a control wild-type strain exposed to 75 mM HU at 37 °C and a cdc22-M45 mutant kept at 36 °C for 4 hours and then exposed to 75 mM HU for the following 3 hours while still at restrictive temperature (right). Images are SUM projections of three central Z slices. Scale bars represent 5 microns. Graph shows the mean ± SD of two independent repetitions of the experiment, and in each repetition at least 100 cells were accounted f [file pbio.3003493.s002.pdf]

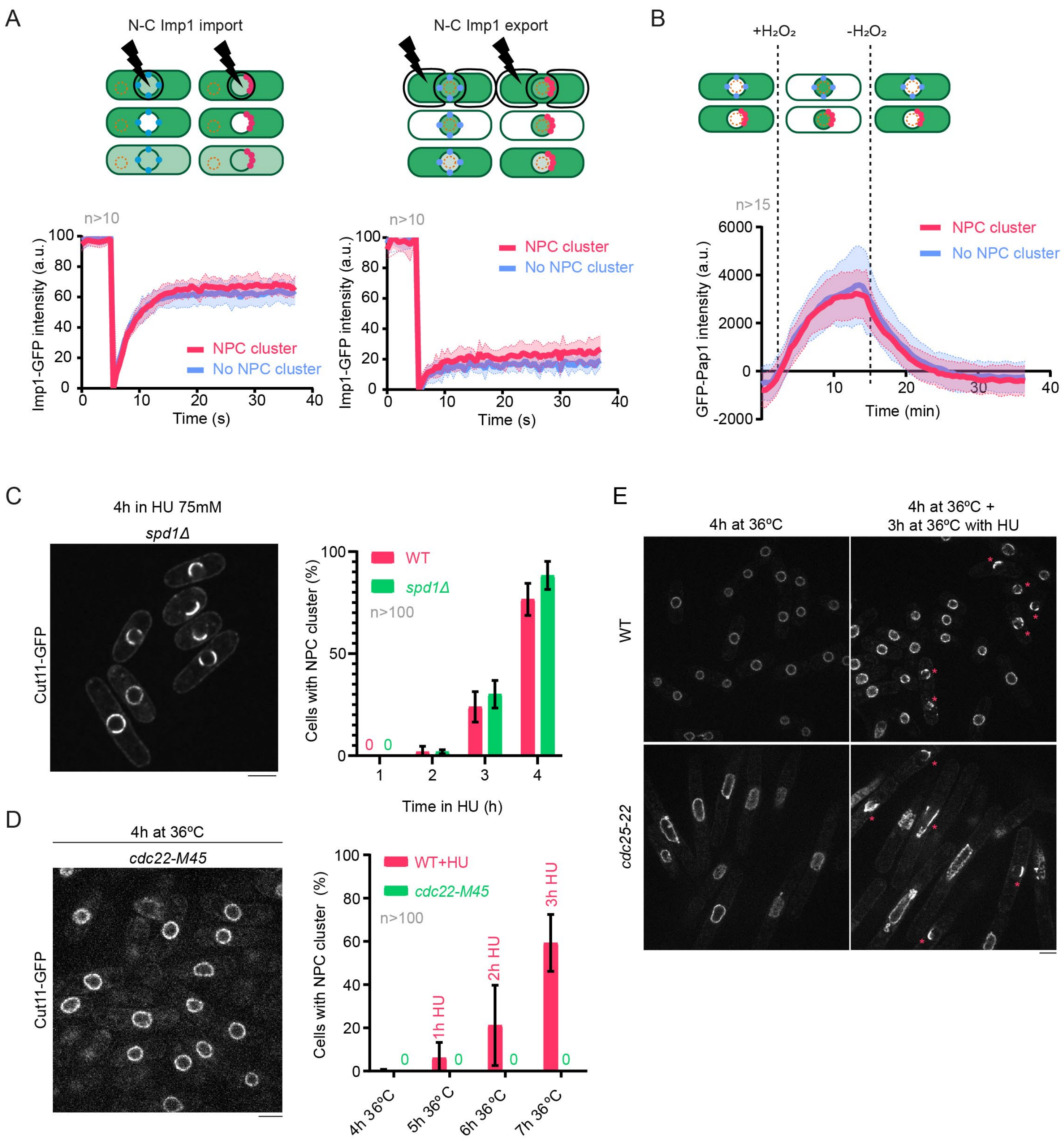

S1 Fig. NPCs clustered after HU treatment remain functional for nucleocytoplasmic transport and their formation is independent of S phase arrest induced by RNR inhibition.

(A) Recovery of importin (Imp1-GFP) fluorescence after photobleaching in the nucleus (left) and in the cytoplasm (right) in cells with and without clustered NPCs exposed to 15 mM HU for 4 hours. Graphs represent the mean ± SD of the fluorescence intensity of Imp1-GFP, normalized to the fluorescence in the indicated compartment right before bleaching, and measured in at least 10 cells of each phenotype. Orange dotted areas mean the placing of the region in which fluorescence intensity is measured. Black dotted areas mean the bleached areas. **(B)** Progression of GFP-Pap1 intensity after exposure and removal of 0.2 mM H_2O_2, in cells with and without clustered NPCs, after a 4-hour exposure to 15 mM HU. Graph represents the mean ± SD of the fluorescence intensity of GFP-Pap1, normalized to the background and measured in at least 15 cells of each phenotype. **(C)** Representative confocal microscopy image of a *spd1Δ* mutant strain after 4 hours in 75 mM HU showing clustered NPCs (left), and graph comparing NPC cluster formation in a wild-type strain and a *spd1Δ* mutant (right). Images are SUM projections of three central Z slices. Scale bars

represent 5 microns. The graph shows the mean ± SD of two independent repetitions of the experiment, and in each repetition at least 100 cells were accounted for each condition. **(D) Left:** Representative confocal microscopy image of a *cdc22-M45* thermosensitive mutant after 4 hours at restrictive temperature (36ºC) proving that these cells do not form NPC clusters *per se* (left), and graph comparing NPC cluster formation in a control wild-type strain exposed to 75 mM HU at 37ºC and a *cdc22-M45* mutant kept at 36ºC for 4 hours and then exposed to 75 mM HU for the following 3 hours while still at restrictive temperature (right). Images are SUM projections of three central Z slices. Scale bars represent 5 microns. Graph shows the mean ± SD of two independent repetitions of the experiment, and in each repetition at least 100 cells were accounted for each condition. **(E)** Representative confocal microscopy images of a wild-type and a *cdc25-22* thermosensitive mutant, both expressing Cut11-GFP, at restrictive temperature (36ºC) after incubation for 4 hours (left) to achieve full blockage of the mutant in the G2/M transition, and after addition of HU for 3 extra hours while kept at 36ºC so cell cycle in the mutant remains blocked (right). When *cdc25-22* cells are blocked in G2/M transition by temperature shift and treated with HU during this blockage, cells form NPC clusters while in G2/M. Magenta asterisks indicate cells with NPC clustering. Images are SUM projections of five central Z slices. Scale bars represent 5 microns. Source data for this figure can be found in S1 Data.
