# Supplementary material for: Hydroxyurea induces an oxidative stress response that triggers ER expansion and cytoplasmic protein aggregation
Source: PLoS Biol. 2025 Nov 19;23(11):e3003493. doi: 10.1371/journal.pbio.3003493 (PMC12654915; doi:10.1371/journal.pbio.3003493)
Supplement: S2 Fig — (A) Quantification of N-Cap reposition time after HU washout following a 4-hour incubation in 75 mM HU, comparing cells with a more expanded ER (‘large ER’; mean area = 4.5 ± 0.4 μm2) versus cells with a less expanded ER (‘small ER’; mean area = 2.5 ± 0.6 μm2). Cells were considered to have a ‘large ER’ when their ER area along the projection of a 3-Z section was over 4 μm2. Graph shows violin plots with the mean ± SD of the reposition time of N-Caps of at least 20 cells with each of the forementioned perinuclear ER morphologies. (B) Timelapse following a group of cells tagged with mCherry-AHDL, previously exposed to 75 mM HU for 4 hours, after drug washout. Cells with a larger ER take longer to recover the even perinuclear architecture than those with a less-expanded ER. Asterisks mark when a cell recovers its normal perinuclear architecture. Images are SUM projections of three central Z slices. Scale bars represent 5 microns. (C-D) Confocal microscopy images of cells expressing INM tags Lem2-GFP and GFP-Ima1 (C), and Man1-GFP (E), opposed to the luminal ER marker mCherry-AHDL, after a 4-hour incubation in 75 mM HU. Magenta arrows show the presence of membranes surrounding the ER lumen. Images are SUM projections of three central Z slices. Scale bars represent 5 microns. (E-F) Transmission electron images (TEM) of a wild-type strain in control conditions (E) and after a 4-hour incubation in 75 mM HU (F). Insets focus on nuclei in each condition; magenta arrows indicate inner nuclear membranes (INM) and outer nuclear membranes continuous with the ER (ONM/ER). N = nucleus; CW, cell wall; NE, nuclear envelope; Cyt. = cytosol. Scale bars represent 1 micrometer, 500 nm or 100 nm, as indicated in each panel. (G) Images of cells expressing mCherry-AHDL and either Cut11-GFP or Ish1-GFP, comparing a wild-type strain with N-Caps to mutant strains cmp7Δ, lem2Δ or vps4Δ, all of which show a phenotype of fragmented ER, after treatment with 75 mM HU for 3 hours. (H) Quantificati [file pbio.3003493.s003.pdf]

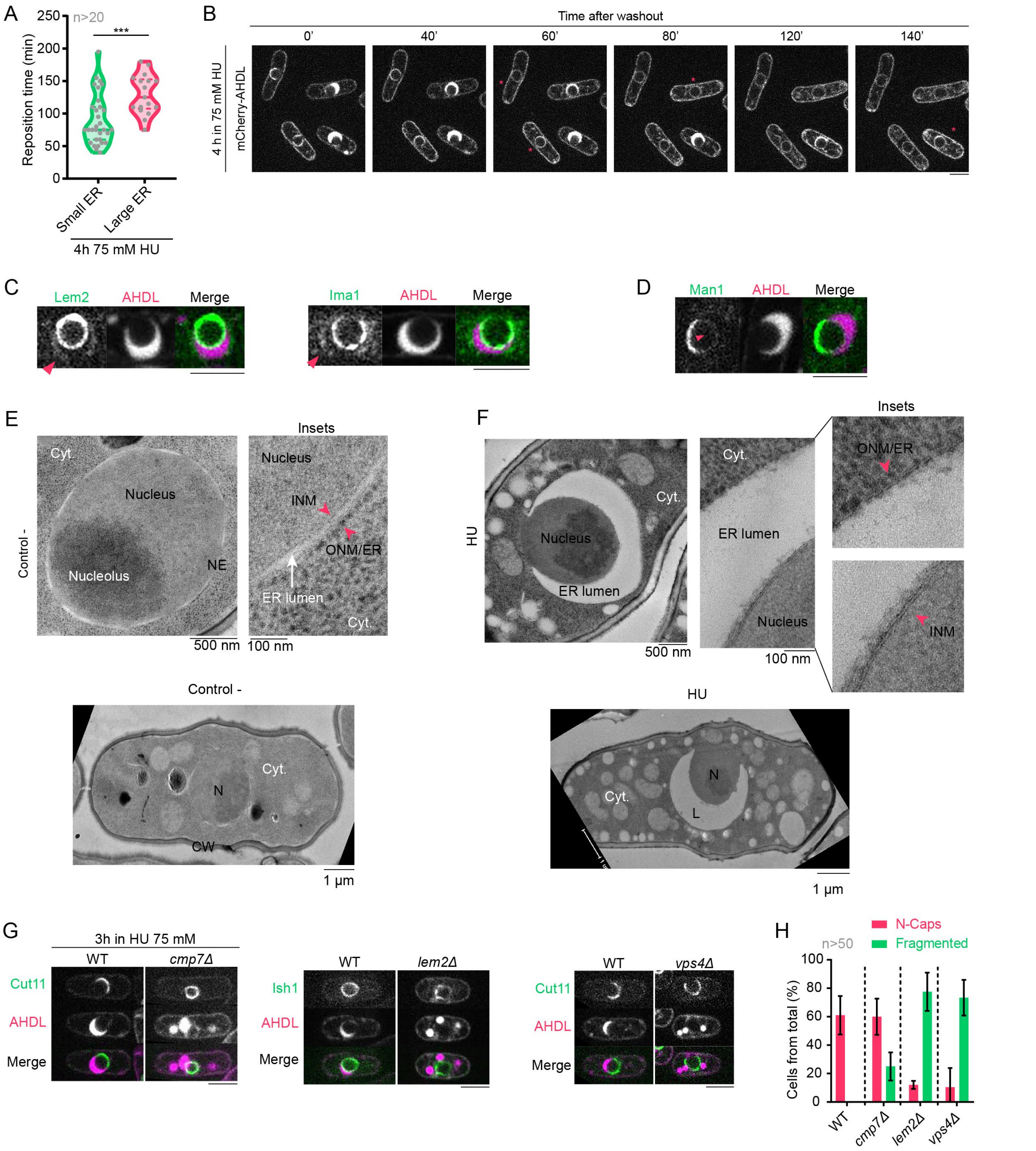

S2 Fig. N-Cap dispersion requires perinuclear ER redistribution, and ER expansion causes INM and ONM/ER membrane separation.

(A) Quantification of N-Cap reposition time after HU washout following a 4-hour incubation in 75 mM HU, comparing cells with a more expanded ER ('large ER'; mean area = 4.5 ± 0.4 µm^2) versus cells with a less expanded ER ('small ER'; mean area = 2.5 ± 0.6 µm^2). Cells were considered to have a 'large ER' when their ER area along the projection of a 3-Z section was over 4 µm^2. Graph shows violin plots with the mean ± SD of the reposition time of N-Caps of at least 20 cells with each of the forementioned perinuclear ER morphologies. **(B)** Timelapse following a group of cells tagged with mCherry-AHDL, previously exposed to

75 mM HU for 4 hours, after drug washout. Cells with a larger ER take longer to recover the even perinuclear architecture than those with a less-expanded ER. Asterisks mark when a cell recovers its normal perinuclear architecture. Images are SUM projections of three central Z slices. Scale bars represent 5 microns. **(C-D)** Confocal microscopy images of cells expressing INM tags Lem2-GFP and GFP-Ima1 (C), and Man1-GFP (E), opposed to the luminal ER marker mCherry-AHDL, after a 4-hour incubation in 75 mM HU. Magenta arrows show the presence of membranes surrounding the ER lumen. Images are SUM projections of three central Z slices. Scale bars represent 5 microns. **(E-F)** Transmission electron images (TEM) of a wild-type strain in control conditions (E) and after a 4-hour incubation in 75 mM HU (F). Insets focus on nuclei in each condition; magenta arrows indicate inner nuclear membranes (INM) and outer nuclear membranes continuous with the ER (ONM/ER). N = nucleus; CW = cell wall; NE = nuclear envelope; Cyt. = cytosol. Scale bars represent 1 micron, 500 nm or 100 nm, as indicated in each panel. **(G)** Images of cells expressing mCherry-AHDL and either Cut11-GFP or Ish1-GFP, comparing a wild-type strain with N-Caps to mutant strains *cmp7Δ*, *lem2Δ* or *vps4Δ*, all of which show a phenotype of fragmented ER, after treatment with 75 mM HU for 3 hours. **(H)** Quantification of the incidence of N-Caps and fragmented ER phenotypes in the total population of cells in the previously addressed conditions. Graphs show the mean ± SD of at least 50 cells in two independent repetitions of the experiment. Source data for this figure can be found in S1 Data.
