# Supplementary material for: Hydroxyurea induces an oxidative stress response that triggers ER expansion and cytoplasmic protein aggregation
Source: PLoS Biol. 2025 Nov 19;23(11):e3003493. doi: 10.1371/journal.pbio.3003493 (PMC12654915; doi:10.1371/journal.pbio.3003493)
Supplement: S3 Fig — (A) Comparison of S. cerevisiae cells expressing mCherry-tagged NUP84 in control conditions (left) and after 3 hours in HU 100mM (right). The asterisk marks a cell with clustered NPCs. Confocal microscopy images are SUM projections of three central Z slices. Scale bar represents 5 µm. (B) In a S. cerevisiae nucleus tagged with NUP84-mCherry, NPCs eventually recover their even distribution along the NE following drug washout after a 3-hour incubation in 100 mM HU. t = time after HU washout. Confocal microscopy images are SUM projections of three central Z slices. Scale bar represents 5 µm. (C) Upper panel: Merge of fluorescence images showing Hoescht 333,248 (blue, nucleus) and ER-ID Red (yellow, endoplasmic reticulum) at 0, 12 and 24 hours post-treatment with HU at 200 µM and 1 mM, DTT 1 mM or the equivalent diluent (control). Scale bars represent 100 µm. Lower panel: Cells were monitored at 0, 12, and 24 hours post Hydroxyurea (HU) treatment, with 3 wells and 5 fields per well counted per time condition. Dyes were applied 45 min before each measurement. ER intensity was segmented to correct for cytoplasmic and extracellular signals, and normalized by ER area in square micrometers (µm²), represented as ‘Total intensity’ (AU × µm²). The control condition (C) corresponds to the highest concentration of HU diluent (water). The number of cells analyzed per experimental condition: 0h: C (959), DTT (1,333), HU 200 µM (1,577), HU 1 mM (1,461). (D) Upper panel: Merge of fluorescence images showing Hoescht 333,248 (blue, nucleus) and ER-ID Red (yellow, ER) at 60 min, 90 min, and 120 min post-treatment with DIA at 50 µM, DTT 100 µM or the equivalent diluent (control), representing the same cells over time. Scale bar represents 100 µm. Lower panel: Cells were monitored from 60 to 120 min after compound and dye addition, segmented to measure ER intensity corrected for cytoplasmic and extracellular surrounding signals and normalized by ER area (in µm²), represented as ‘Total int [file pbio.3003493.s004.pdf]

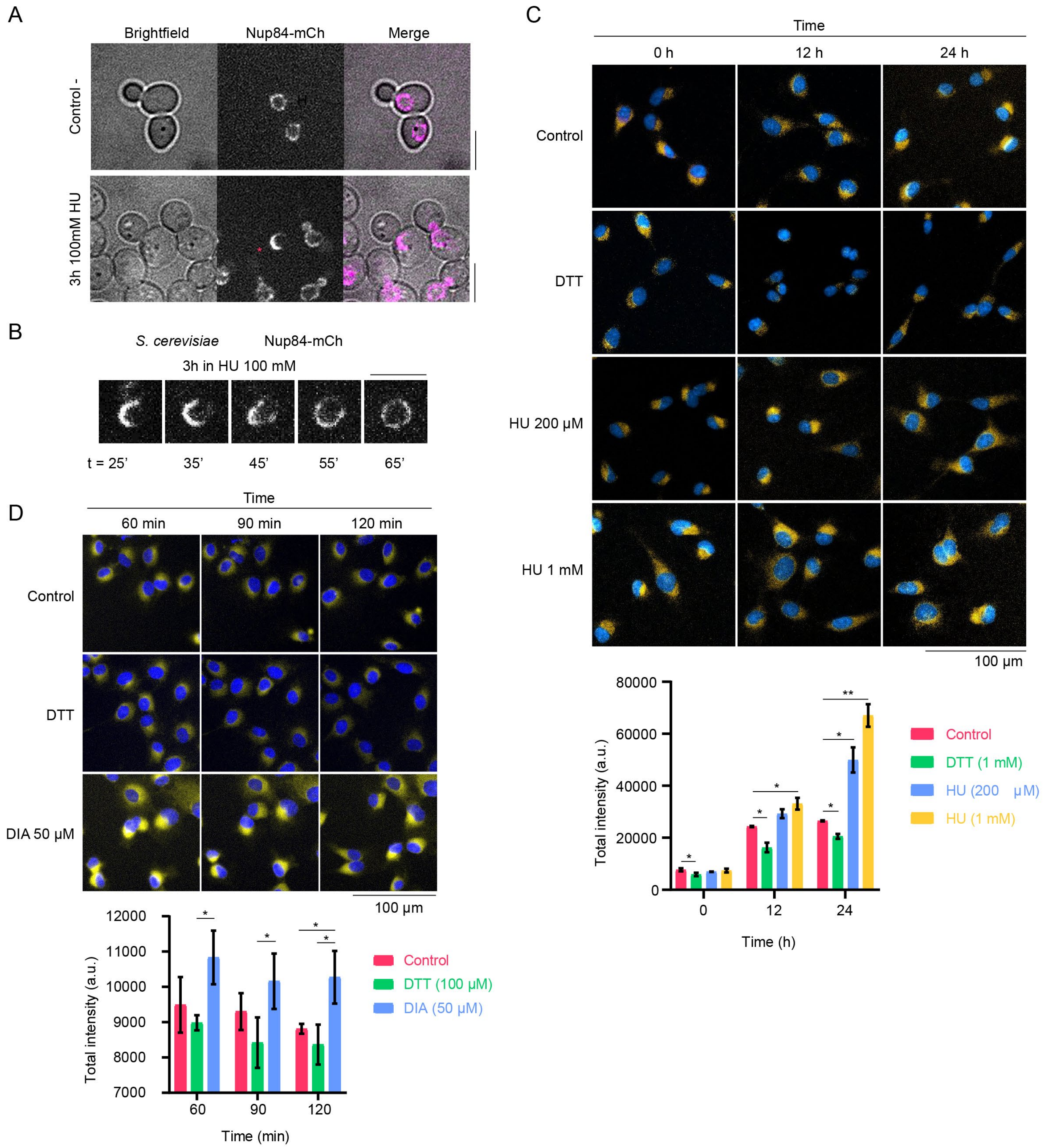

S3 Fig. HU-induced perinuclear architecture alterations are evolutionarily conserved.

(A) Comparison of *S. cerevisiae* cells expressing mCherry-tagged NUP84 in control conditions (left) and after 3 hours in HU 100mM (right). The asterisk marks a cell with clustered NPCs. Confocal microscopy images are SUM projections of three central Z slices. Scale bar represents 5 μm. **(B)** In a *S. cerevisiae* nucleus tagged with NUP84-mCherry, NPCs eventually recover their even distribution along the NE following drug washout after a 3-hour incubation in 100 mM HU. t = time after HU washout. Confocal microscopy images are SUM projections of three central Z slices. Scale bar represents 5 μm. **(C) Upper panel:** Merge of fluorescence images showing Hoescht 333248 (blue, nucleus) and ER-ID Red (yellow, endoplasmic reticulum) at 0, 12 and 24 hours post-treatment with HU at 200 μM and 1 mM, DTT 1 mM or the equivalent diluent (control). Scale bars represent 100 μm. **Lower panel:** Cells were monitored at 0, 12, and 24 hours

post Hydroxyurea (HU) treatment, with 3 wells and 5 fields per well counted per time condition. Dyes were applied 45 minutes before each measurement. ER intensity was segmented to correct for cytoplasmic and extracellular signals, and normalized by ER area in square micrometers (μm²), represented as 'Total intensity' (AU×μm²). The control condition (C) corresponds to the highest concentration of HU diluent (water). The number of cells analyzed per experimental condition: 0h: C (959), DTT (1333), HU 200 μM (1577), HU 1 mM (1461). **(D) Upper panel:** Merge of fluorescence images showing Hoescht 333248 (blue, nucleus) and ER-ID Red (yellow, ER) at 60 min, 90 min, and 120 min post-treatment with DIA at 50 μM, DTT 100 μM or the equivalent diluent (control), representing the same cells over time. Scale bar represents 100 μm. **Lower panel:** Cells were monitored from 60 to 120 minutes after compound and dye addition, segmented to measure ER intensity corrected for cytoplasmic and extracellular surrounding signals and normalized by ER area (in μm²), represented as 'Total intensity' (AU×μm²). The number of cells analyzed per experimental condition was derived from three independent wells: DIA: Treatment with Diamide (1335 cells). Control: Control group receiving equivalent concentrations of diluents (1792 cells). DTT: Treatment with DTT (1377 cells). Source data for this figure can be found in S1 Data.
