# Supplementary material for: Hydroxyurea induces an oxidative stress response that triggers ER expansion and cytoplasmic protein aggregation
Source: PLoS Biol. 2025 Nov 19;23(11):e3003493. doi: 10.1371/journal.pbio.3003493 (PMC12654915; doi:10.1371/journal.pbio.3003493)
Supplement: S4 Fig — (A) FRAP experiment consisting of photobleaching of the perinuclear ER to measure Bip1-GFP fluorescence recovery either in untreated conditions, in 3 mM DIA after a 4-hour incubation, or in 75 mM HU also after a 4-hour incubation. Cells with expanded ER (N-Cap) showed almost no fluorescence recovery in comparison with cells without N-Cap and in control conditions. The graph represents the mean ± SD of the fluorescence intensity of Imp1-GFP, normalized to the fluorescence in the indicated compartment right before bleaching, and measured in at least 10 cells of each phenotype. (B) FRAP experiment consisting of photobleaching half of the perinuclear ER to measure Bip1-GFP fluorescence recovery in both sides of the compartment. The graph represents the mean ± SD of the fluorescence intensity of Imp1-GFP, normalized to the fluorescence in the indicated compartment right before bleaching, and measured in at least 10 cells of each phenotype. (C) Confocal microscopy images of cells expressing Vgl1-GFP and Hsp104-mRFP in untreated conditions and after 4 hours in 75 mM HU or 3 mM DIA. Insets below show individual Hsp104-mRFP aggregates and show that Vgl1-GFP is not part of the aggregate. (D) Images of cells expressing Hsp104-GFP and Ish1-mScarlet in untreated conditions and after 4 hours in 75 mM HU or 3 mM DIA. Insets below show that Hsp104 aggregates are not surrounded by Ish1-containing membrane. (E-F) Images of cells expressing Hsp104-mRFP and either Rtn1-GFP (E) or Yop1-GFP (F) in untreated conditions and after 4 hours in either 75 mM HU or 3 mM DIA. Insets below show that Hsp104-containing aggregates are not surrounded by transmembrane ER proteins. (G) Images of cells expressing Gma12-GFP and Hsp104-mRFP in untreated conditions and after 4 hours in either 75 mM HU or 3 mM DIA. Insets on the right show that Hsp104-mRFP aggregates do not colocalize with Gma12-GFP foci. Confocal microscopy images are SUM projections of 3 central Z slices. Scale bar represents 5 μm. Source [file pbio.3003493.s005.pdf]

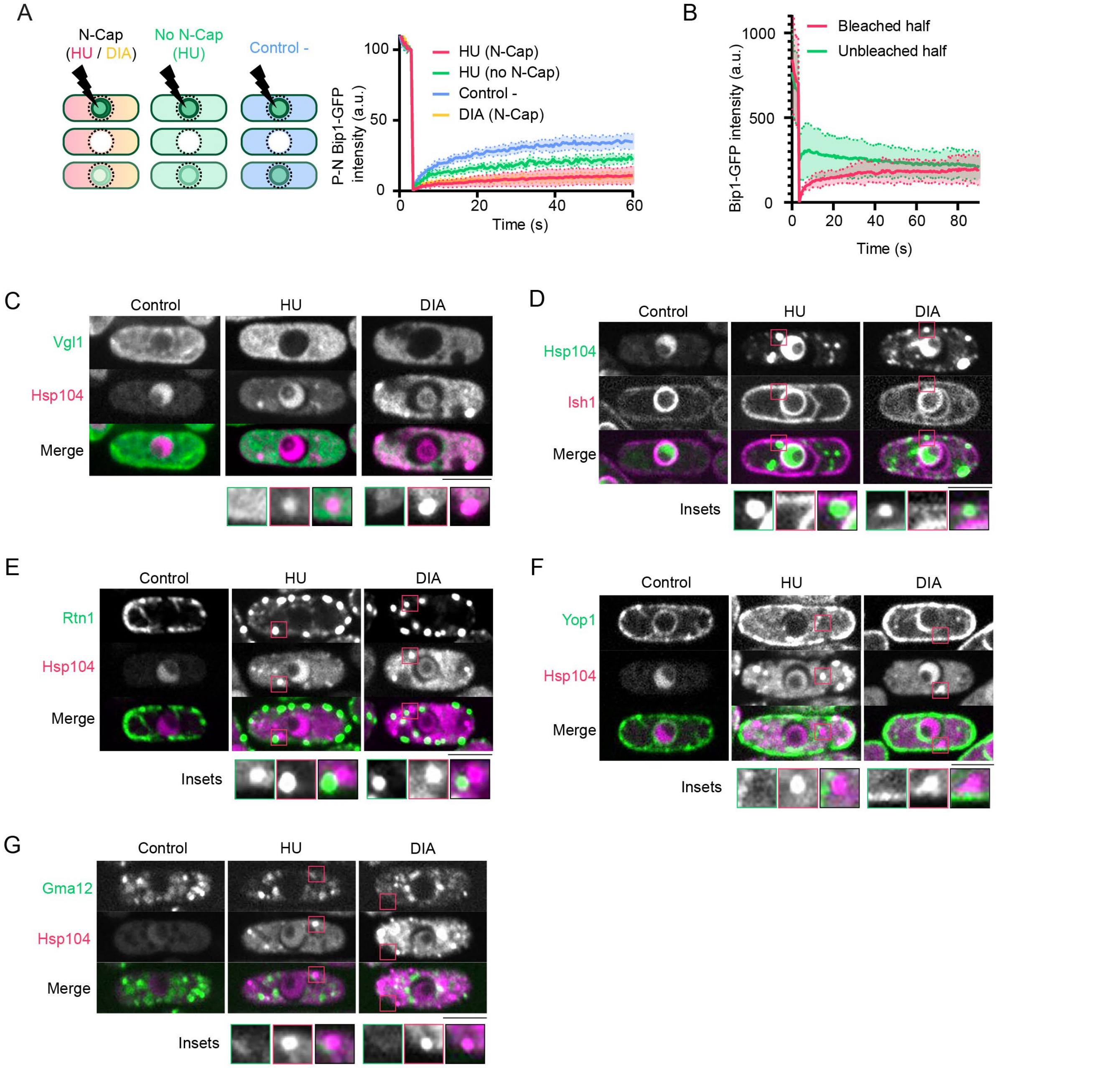

S4 Fig. Bip1-GFP mobility is reduced during HU or DIA treatments, and cytoplasmic foci are not membrane-bound nor correspond to canonical heat shock induced stress granules.

(A) FRAP experiment consisting of photobleaching of the perinuclear ER to measure Bip1-GFP fluorescence recovery either in untreated conditions, in 3 mM DIA after a 4-hour incubation, or in 75 mM HU also after a 4-hour incubation. Cells with expanded ER (N-Cap) showed almost no fluorescence recovery in comparison with cells without N-Cap and in control conditions. The graph represents the mean ± SD of the fluorescence intensity of Imp1-GFP, normalized to the fluorescence in the indicated compartment right before bleaching, and measured in at least 10 cells of each phenotype. **(B)** FRAP experiment consisting of photobleaching half of the perinuclear ER to measure Bip1-GFP fluorescence recovery in both sides of the compartment. The graph represents the mean ± SD of the fluorescence intensity of Imp1-GFP, normalized to the fluorescence in the indicated compartment right before bleaching, and measured in at least 10 cells of each phenotype. **(C)** Confocal microscopy images of cells expressing Vgl1-GFP and Hsp104-mRFP in untreated conditions and after 4 hours in 75 mM HU or 3 mM DIA. Insets below show individual Hsp104-mRFP aggregates and show that Vgl1-GFP is not part of the aggregate. **(D)** Images of cells expressing Hsp104-GFP and Ish1-

mScarlet in untreated conditions and after 4 hours in 75 mM HU or 3 mM DIA. Insets below show that Hsp104 aggregates are not surrounded by Ish1-containing membrane. **(E-F)** Images of cells expressing Hsp104-mRFP and either Rtn1-GFP (E) or Yop1-GFP (F) in untreated conditions and after 4 hours in either 75 mM HU or 3 mM DIA. Insets below show that Hsp104-containing aggregates are not surrounded by transmembrane ER proteins. **(G)** Images of cells expressing Gma12-GFP and Hsp104-mRFP in untreated conditions and after 4 hours in either 75 mM HU or 3 mM DIA. Insets on the right show that Hsp104-mRFP aggregates do not colocalize with Gma12-GFP foci.

Confocal microscopy images are SUM projections of 3 central Z slices. Scale bar represents 5 µm. Source data for this figure can be found in S1 Data.
