# Supplementary material for: Hydroxyurea induces an oxidative stress response that triggers ER expansion and cytoplasmic protein aggregation
Source: PLoS Biol. 2025 Nov 19;23(11):e3003493. doi: 10.1371/journal.pbio.3003493 (PMC12654915; doi:10.1371/journal.pbio.3003493)
Supplement: S5 Fig — (A) Quantification of the incidence of the N-Cap phenotype in a wild-type strain during 3 mM DIA treatment (upper graph) or 75 mM HU treatment (lower graph) when combined with 100 μg/mL anisomycin (ANM). Graphs show the mean ± SD of two independent repetitions of the experiment, and in each repetition at least 100 cells were accounted for each condition. (B) Confocal microscopy images of cells expressing Hsp104-GFP (green) and mCherry-AHDL (magenta) after a 3-hour incubation in either control conditions, 100 μg/mL ANM, 3 mM DIA, 75 mM HU, DIA and ANM, or HU and ANM. Hsp104-GFP aggregates are detected only in DIA and HU treatments. Images are SUM projections of 3 central Z slices. Scale bars represent 5 μm. Source data for this figure can be found in S1 Data. (PDF) [file pbio.3003493.s006.pdf]

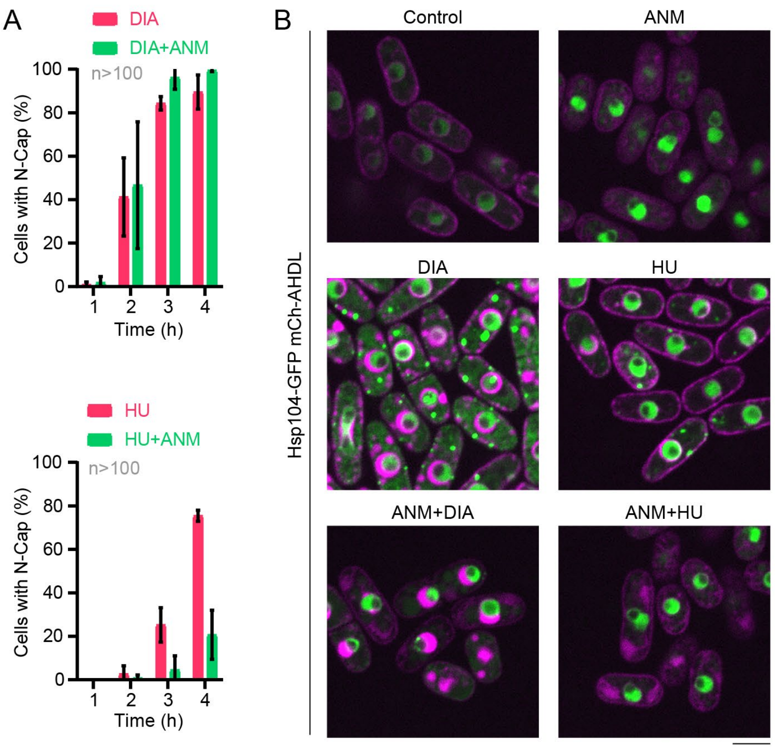

S5 Fig: Anisomycin recapitulates CHX behavior when combined with HU or DIA

(A) Quantification of the incidence of the N-Cap phenotype in a wild-type strain during 3 mM DIA treatment (upper graph) or 75 mM HU treatment (lower graph) when combined with 100 µg/mL anisomycin (ANM). Graphs show the mean ± SD of two independent repetitions of the experiment, and in each repetition at least 100 cells were accounted for each condition. **(B)** Confocal microscopy images of cells expressing Hsp104-GFP (green) and mCherry-AHDL (magenta) after a 3-hour incubation in either control conditions, 100 µg/mL ANM, 3 mM DIA, 75 mM HU, DIA and ANM, or HU and ANM. Hsp104-GFP aggregates are detected only in DIA and HU treatments. Images are SUM projections of 3 central Z slices. Scale bars represent 5 µm. Source data for this figure can be found in S1 Data.
