# Supplementary material for: Hydroxyurea induces an oxidative stress response that triggers ER expansion and cytoplasmic protein aggregation
Source: PLoS Biol. 2025 Nov 19;23(11):e3003493. doi: 10.1371/journal.pbio.3003493 (PMC12654915; doi:10.1371/journal.pbio.3003493)
Supplement: S6 Fig — (A) Canonical ER stress as seen with the luminal ER tag mCherry-AHDL in a wild-type strain after treatment with DTT 2 mM and 5 mM, and with tunicamycin (Tn) 0.6 μg/mL and 2 μg/mL, after a 2-hour incubation. Images are SUM projections of three central Z slices. Scale bars represent 5 microns. (B) Upper panels: Confocal microscopy images of a representative cell of a wild-type strain and an ire1Δ strain, both expressing Cut11-GFP and mCherry-AHDL and showing N-Caps after incubation in 3 mM DIA for 4 hours. Images are SUM projections of three central Z slices. Scale bars represent 5 microns. Lower panels: Quantification of the incidence of the N-Cap phenotype in a wild-type strain and an ire1Δ strain during 3 mM DIA treatment. Graph shows the mean ± SD of three independent repetitions of the experiment, and in each repetition at least 100 cells were accounted for each condition. (C) Viability assay of a wild-type strain and an ire1Δ strain in control conditions, in presence of 2.5 mM DTT and in 3 mM DIA after a 2-day incubation at 30 °C. (D) Principal component analysis separating the three independent repetitions of each of the five conditions addressed in the RNA-Seq. Replicates are very consistent, and HU and DIA conditions are well-differentiated between them and with respect to the control. (E) Heatmap with the expression profile of upregulated and downregulated genes in 3 mM DIA after 30 and 60 min, and in 75 mM HU after 60 and 150 min, with respect to an untreated culture. Three replicates of each condition were assessed. Blue indicates upregulation; yellow indicates downregulation; LFC = Log2 fold change. (F) Volcano plots detailing the significantly upregulated and downregulated genes (LFC ≥ 1 or LFC ≤ −1 respectively, p-value<0.05) in DIA (upper graph) and HU (lower graph), pooling together both times addressed. Red dots show genes that surpass the set thresholds of LFC and p-value. Highlighted genes are some of the common genes in all conditions; ‘variables’ [file pbio.3003493.s007.pdf]

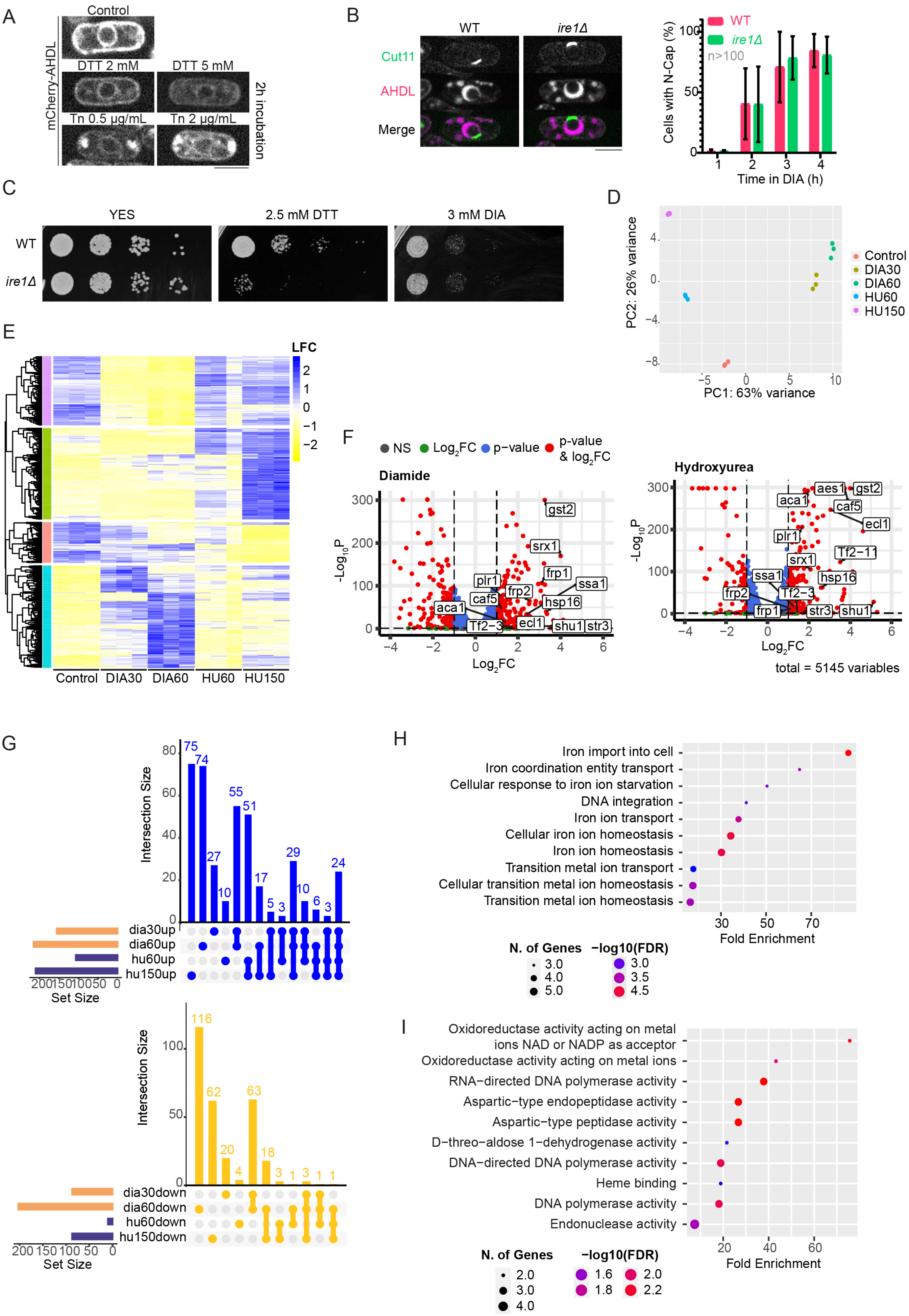
A
Control
mCherry-AHDL
DTT 2 mM
DTT 5 mM
Tn 0.5 μg/mL
Tn 2 μg/mL
2h incubation
B
WT
ire1Δ
Cut11
AHDL
Merge
Cells with N-Cap (%)
WT
ire1Δ
n>100
Time in DIA (h)
C
YES
2.5 mM DTT
3 mM DIA
WT
ire1Δ
D
PC2: 26% variance
PC1: 63% variance
Control
DIA30
DIA60
HU60
HU150
E
LFC
Control
DIA30
DIA60
HU60
HU150
F
NS
Log2FC
p-value
p-value & log2FC
Diamide
Hydroxyurea
-Log10P
Log2FC
total = 5145 variables
gst2
srx1
frp1
ssa1
plr1
frp2
caf5
hsp16
aca1
Tf2-3
ecl1
shu1
str3
aes1
Tf2-11
G
Intersection Size
Set Size
dia30up
dia60up
hu60up
hu150up
dia30down
dia60down
hu60down
hu150down
H
Iron import into cell
Iron coordination entity transport
Cellular response to iron ion starvation
DNA integration
Iron ion transport
Cellular iron ion homeostasis
Iron ion homeostasis
Transition metal ion transport
Cellular transition metal ion homeostasis
Transition metal ion homeostasis
Fold Enrichment
N. of Genes
−log10(FDR)
I
Oxidoreductase activity acting on metal ions NAD or NADP as acceptor
Oxidoreductase activity acting on metal ions
RNA−directed DNA polymerase activity
Aspartic−type endopeptidase activity
Aspartic−type peptidase activity
D−threo−aldose 1−dehydrogenase activity
DNA−directed DNA polymerase activity
Heme binding
DNA polymerase activity
Endonuclease activity
N. of Genes
−log10(FDR)
Fold Enrichment

S6 Fig. HU and DIA induce a UPR-independent response.

(A) Canonical ER stress as seen with the luminal ER tag mCherry-AHDL in a wild-type strain after treatment with DTT 2 mM and 5 mM, and with tunicamycin (Tn) 0.6 µg/mL and 2 µg/mL, after a 2-hour incubation. Images are SUM projections of three central Z slices. Scale bars represent 5 microns. **(B) Upper panels:** Confocal microscopy images of a representative cell of a wild-type strain and an *ire1Δ* strain, both expressing Cut11-GFP and mCherry-AHDL and showing N-Caps after incubation in 3 mM DIA for 4 hours. Images are SUM projections of three central Z slices. Scale bars represent 5 microns. **Lower panels:** Quantification of the incidence of the N-Cap phenotype in a wild-type strain and an *ire1Δ* strain during 3 mM DIA treatment. Graph shows the mean ± SD of three independent repetitions of the experiment, and in each repetition at least 100 cells were accounted for each condition. **(C)** Viability assay of a wild-type strain and an *ire1Δ* strain in control conditions, in presence of 2.5 mM DTT and in 3 mM DIA after a 2-day incubation at 30ºC. **(D)** Principal component analysis separating the three independent repetitions of each of the five conditions addressed in the RNA-Seq. Replicates are very consistent, and HU and DIA conditions are well-differentiated between them and with respect to the control. **(E)** Heatmap with the expression profile of upregulated and downregulated genes in 3 mM DIA after 30 and 60 minutes, and in 75 mM HU after 60 and 150 minutes, with respect to an untreated culture. Three replicates of each condition were assessed. Blue indicates upregulation; yellow indicates downregulation; LFC = Log2 fold change. **(F)** Volcano plots detailing the significantly upregulated and downregulated genes (LFC≥1 or LFC≤-1 respectively, p-value<0.05) in DIA (upper graph) and HU (lower graph), pooling together both times addressed. Red dots show genes that surpass the set thresholds of LFC and p-value. Highlighted genes are some of the common genes in all conditions; 'variables' stands for the total number of genes. **(G)** Upset plots for comparing genes that are upregulated (upper graph) and downregulated (lower graph) between the four treatment conditions. The numbers indicate the genes that are common to the designated combination of conditions. In the case of the unconnected dots, numbers show genes exclusive to that condition alone. Set size graphs indicate the total number of genes in each condition. **(H)** GO term enrichment for the biological processes shared among the genes common to the four treatment conditions. **(I)** GO term enrichment for the molecular functions shared among the genes common to the four treatment conditions. Source data for this figure can be found in S1 Data. Raw data from the RNA-Seq analysis is publicly available in *Gene Expression Omnibus* under the accession number GSE309439.
