# Supplementary material for: Hydroxyurea induces an oxidative stress response that triggers ER expansion and cytoplasmic protein aggregation
Source: PLoS Biol. 2025 Nov 19;23(11):e3003493. doi: 10.1371/journal.pbio.3003493 (PMC12654915; doi:10.1371/journal.pbio.3003493)
Supplement: S1 Table — Quantification of the total amount of proteins and peptide groups detected in each of the three experimental conditions (C-: untreated control; DIA: 3-hour incubation in 3 mM DIA; HU: 3-hour incubation in 75 mM HU). (PDF) [file pbio.3003493.s008.pdf]

**S1 Table: HU and DIA induce protein glutathionylation**

Quantification of the total amount of proteins and peptide groups detected in each of the three experimental conditions (C-: untreated control; DIA: 3-hour incubation in 3 mM DIA; HU: 3-hour incubation in 75 mM HU).

| | | | **GLUTATHIONYLATED** | |
|---|---|---|---|---|
| | **TOTAL PROTEINS** | **TOTAL PEPTIDIC GROUPS** | **PROTEINS** | **PEPTIDIC GROUPS** |
| **Control** | 2099 | 12680 | 5 | 6 |
| **HU** | 2433 | 17931 | 25 | 29 |
| **DIA** | 2345 | 15773 | 14 | 14 |
