# Supplementary material for: Hydroxyurea induces an oxidative stress response that triggers ER expansion and cytoplasmic protein aggregation
Source: PLoS Biol. 2025 Nov 19;23(11):e3003493. doi: 10.1371/journal.pbio.3003493 (PMC12654915; doi:10.1371/journal.pbio.3003493)
Supplement: S2 Table — Summary of the proteins detected as glutathionylated by mass spectrometry after a 3-hour treatment with 75 mM HU. Sum PEP Score represents the protein score, calculated as the sum of the negative logarithm of the PEP values of the connected PSMs. The PEP (Posterior Error Probability) indicates the probability that a reported match is a random event (higher values indicate greater confidence in identification). Coverage (%) refers to the sequence coverage percentage, calculated by dividing the number of amino acids in all identified peptides by the total number of amino acids in the full protein sequence (higher values indicate better coverage). (PDF) [file pbio.3003493.s009.pdf]

S2 Table: Proteins glutathionylated in HU

Summary of the proteins detected as glutathionylated by mass spectrometry after a 3-hour treatment with 75 mM HU. Sum PEP Score represents the protein score, calculated as the sum of the negative logarithm of the PEP values of the connected PSMs. The PEP (Posterior Error Probability) indicates the probability that a reported match is a random event (higher values indicate greater confidence in identification). Coverage (%) refers to the sequence coverage percentage, calculated by dividing the number of amino acids in all identified peptides by the total number of amino acids in the full protein sequence (higher values indicate better coverage).

SYSTEMATIC ID	GENE NAME	PRODUCT DESCRIPTION	COVERAGE [%]	Sum PEP Score
**SPAC13G7.02c**	*ssa1*	Hsp70 family heat shock protein Ssa1	49	336.397
**SPAC144.11**	*rps1102*	40S ribosomal protein S11	67	121.451
**SPAC1F8.07c**	*pdc101*	pyruvate decarboxylase	46	441.977
**SPAC22A12.04c**	*rps2201*	40S ribosomal protein S15a	70	75.941
**SPAC23A1.08c**	*rpl3401*	60S ribosomal protein L34	68	54.599
**SPAC23A1.10**	*tef102*	translation elongation factor EF-1 alpha Ef1a-b	53	363.504
**SPAC26A3.07c**	*rpl1101*	60S ribosomal protein L11	72	87.199
**SPAC3H5.12c**	*rpl501*	60S ribosomal protein L5	60	161.17
**SPAC4H3.10c**	*pyk1*	pyruvate kinase	69	501.101
**SPAC6B12.15**	*cpc2*	ribosome-associated signalling scaffold, receptor of activated C kinase (RACK1) ortholog Cpc2	80	196.224
**SPAC9.09**	*met26*	homocysteine methyltransferase Met26	62	463.773
**SPAC926.04c**	*hsp90*	Hsp90 chaperone	48	466.869
**SPBC11C11.09c**	*rpl502*	60S ribosomal protein L5	60	161.17
**SPBC15C4.04c**		amino acid/polyamine family transmembrane transporter	8	7.879
**SPBC1815.01**	*eno101*	enolase	62	385.258
**SPBC215.05**	*gpd1*	glycerol-3-phosphate dehydrogenase Gpd1	59	197.91
**SPBC32F12.11**	*tdh1*	glyceraldehyde-3-phosphate dehydrogenase Tdh1	71	462.798
**SPBC354.12**	*gpd3*	glyceraldehyde 3-phosphate dehydrogenase Gpd3	64	305.903

**SPCC1322.15**	*rpl3402*	60S ribosomal protein L34	61	50.94
**SPCC13B11.01**	*adh1*	alcohol dehydrogenase Adh1	68	357.578
**SPCC1494.07**	*trm732*	tRNA (cytosine 32-2'-O)-methyltransferase regulator Trm732	2	8.884
**SPCC1739.13**	*ssa2*	Hsp70 family heat shock protein Ssa2	64	597.2
**SPCC576.08c**	*rps2*	40S ribosomal protein S2	40	144.729
**SPCC622.18**	*rpl6*	60S ribosomal protein L6	65	98.093
**SPCC794.09c**	*tef101*	translation elongation factor EF-1 alpha Ef1a-a	53	363.504
