# Supplementary material for: Hydroxyurea induces an oxidative stress response that triggers ER expansion and cytoplasmic protein aggregation
Source: PLoS Biol. 2025 Nov 19;23(11):e3003493. doi: 10.1371/journal.pbio.3003493 (PMC12654915; doi:10.1371/journal.pbio.3003493)
Supplement: S3 Table — Summary of the proteins detected as glutathionylated by mass spectrometry after a 3-hour treatment with 3 mM DIA. Sum PEP Score represents the protein score, calculated as the sum of the negative logarithm of the PEP values of the connected PSMs. The PEP (Posterior Error Probability) indicates the probability that a reported match is a random event (higher values indicate greater confidence in identification). Coverage (%) refers to the sequence coverage percentage, calculated by dividing the number of amino acids in all identified peptides by the total number of amino acids in the full protein sequence (higher values indicate better coverage). (PDF) [file pbio.3003493.s010.pdf]

**S3 Table: Proteins glutathionylated in DIA**

Summary of the proteins detected as glutathionylated by mass spectrometry after a 3-hour treatment with 3 mM DIA. Sum PEP Score represents the protein score, calculated as the sum of the negative logarithm of the PEP values of the connected PSMs. The PEP (Posterior Error Probability) indicates the probability that a reported match is a random event (higher values indicate greater confidence in identification). Coverage (%) refers to the sequence coverage percentage, calculated by dividing the number of amino acids in all identified peptides by the total number of amino acids in the full protein sequence (higher values indicate better coverage).

| SYSTEMATIC ID | GENE NAME | PRODUCT DESCRIPTION | COVERAGE [%] | SUM PEP SCORE |
|---|---|---|---|---|
| **SPAC1F8.07c** | *pdc101* | pyruvate decarboxylase | 42 | 389.309 |
| **SPAC20G8.04c** | *cir2* | mitochondrial electron transfer flavoprotein-ubiquinone oxidoreductase Cir2 | 23 | 41.925 |
| **SPAC23A1.08c** | *rpl3401* | 60S ribosomal protein L34 | 56 | 37.227 |
| **SPAC23A1.10** | *tef102* | translation elongation factor EF-1 alpha Ef1a-b | 51 | 313.833 |
| **SPAC26A3.07c** | *rpl1101* | 60S ribosomal protein L11 | 53 | 64.405 |
| **SPAC4H3.10c** | *pyk1* | pyruvate kinase | 62 | 367.646 |
| **SPAC9.09** | *met26* | homocysteine methyltransferase Met26 | 57 | 432.846 |
| **SPAC926.04c** | *hsp90* | Hsp90 chaperone | 48 | 454.509 |
| **SPBC1709.02c** | *vrs1* | cytoplasmic valine-tRNA ligase Vrs1/Vas1 | 43 | 251.773 |
| **SPBC17G9.07** | *rps2402* | 40S ribosomal protein S24 | 51 | 90.797 |
| **SPBC1815.01** | *eno101* | enolase | 57 | 327.552 |
| **SPCC1322.15** | *rpl3402* | 60S ribosomal protein L34 | 57 | 37.227 |
| **SPCC576.08c** | *rps2* | 40S ribosomal protein S2 | 45 | 144.642 |
| **SPCC794.09c** | *tef101* | translation elongation factor EF-1 alpha Ef1a-a | 51 | 313.833 |
