# Supplementary material for: Hydroxyurea induces an oxidative stress response that triggers ER expansion and cytoplasmic protein aggregation
Source: PLoS Biol. 2025 Nov 19;23(11):e3003493. doi: 10.1371/journal.pbio.3003493 (PMC12654915; doi:10.1371/journal.pbio.3003493)
Supplement: S4 Table — Summary of the proteins detected as glutathionylated by mass spectrometry in an untreated culture. Sum PEP Score represents the protein score, calculated as the sum of the negative logarithm of the PEP values of the connected PSMs. The PEP (Posterior Error Probability) indicates the probability that a reported match is a random event (higher values indicate greater confidence in identification). Coverage (%) refers to the sequence coverage percentage, calculated by dividing the number of amino acids in all identified peptides by the total number of amino acids in the full protein sequence (higher values indicate better coverage). (PDF) [file pbio.3003493.s011.pdf]

S4 Table: Proteins glutathionylated in untreated conditions

Summary of the proteins detected as glutathionylated by mass spectrometry in an untreated culture. Sum PEP Score represents the protein score, calculated as the sum of the negative logarithm of the PEP values of the connected PSMs. The PEP (Posterior Error Probability) indicates the probability that a reported match is a random event (higher values indicate greater confidence in identification). Coverage (%) refers to the sequence coverage percentage, calculated by dividing the number of amino acids in all identified peptides by the total number of amino acids in the full protein sequence (higher values indicate better coverage).

SYSTEMATIC ID	GENE NAME	PRODUCT DESCRIPTION	COVERAGE [%]	SUM PEP SCORE
**SPAC1F8.07c**	*pdc101*	pyruvate decarboxylase	42	347.484
**SPBC17D11.05**	*tif301*	translation initiation factor eIF3a	36	227.643
**SPBC1815.01**	*eno101*	enolase	50	296.52
**SPCC290.03c**	*nup186*	nucleoporin Nup186	3	10.624
**SPCC576.08c**	*rps2*	40S ribosomal protein S2	40	120.978
