# Supplementary material for: Hydroxyurea induces an oxidative stress response that triggers ER expansion and cytoplasmic protein aggregation
Source: PLoS Biol. 2025 Nov 19;23(11):e3003493. doi: 10.1371/journal.pbio.3003493 (PMC12654915; doi:10.1371/journal.pbio.3003493)
Supplement: S5 Table — Proteins common to both HU and DIA treatment, with their detailed cellular localization and involvement in biological pathways. (PDF) [file pbio.3003493.s012.pdf]

S5 Table: Summary of proteins glutathionylated both in HU and DIA

Proteins common to both HU and DIA treatment, with their detailed cellular localization and involvement in biological pathways.

SYSTEMATIC ID	GENE NAME	PRODUCT DESCRIPTION	CELLULAR LOCALIZATION	INVOLVEMENT IN BIOLOGICAL PATHWAYS
**SPCC576.08c**	*rps2*	40S ribosomal protein S2	Cytosol	Cytoplasmic translation, ribosome assembly
**SPAC26A3.07c**	*rpl1101*	60S ribosomal protein L11	Cytosol	Cytoplasmic translation, ribosome assembly
**SPAC23A1.08c**	*rpl3401*	60S ribosomal protein L34	Cytosol	Cytoplasmic translation, ribosome structural constituent
**SPCC1322.15**	*rpl3402*	60S ribosomal protein L34	Cytosol, Nucleolus	Cytoplasmic translation, ribosome biogenesis, ribosome structural constituent
**SPBC1815.01**	*eno101*	Enolase	Cytosol, Nucleus	Glycolysis
**SPAC9.09**	*met26*	Homocysteine methyltransferase	Cytosol, Nucleus	Methionine biosynthesis
**SPAC926.04c**	*hsp90*	Heat shock protein 90 homolog	Cytosol, Mitochondrion, etc.	Protein folding, stress response
**SPAC1F8.07c**	*pdc101*	Probable pyruvate decarboxylase	Cytosol	Fermentation, aromatic amino acid catabolism
**SPAC4H3.10c**	*pyk1*	Pyruvate kinase	Cytosol	Glycolysis
**SPCC794.09c**	*tef101*	Translation elongation factor EF-1 alpha	Cytosol	Cytoplasmic translational elongation
**SPAC23A1.10**	*tef102*	Translation elongation factor EF-1 alpha	Cytosol	Cytoplasmic translational elongation
