# Supplementary material for: Hydroxyurea induces an oxidative stress response that triggers ER expansion and cytoplasmic protein aggregation
Source: PLoS Biol. 2025 Nov 19;23(11):e3003493. doi: 10.1371/journal.pbio.3003493 (PMC12654915; doi:10.1371/journal.pbio.3003493)
Supplement: S1 Raw Images — (PDF) [file pbio.3003493.s013.pdf]

Stain-free (loading control)

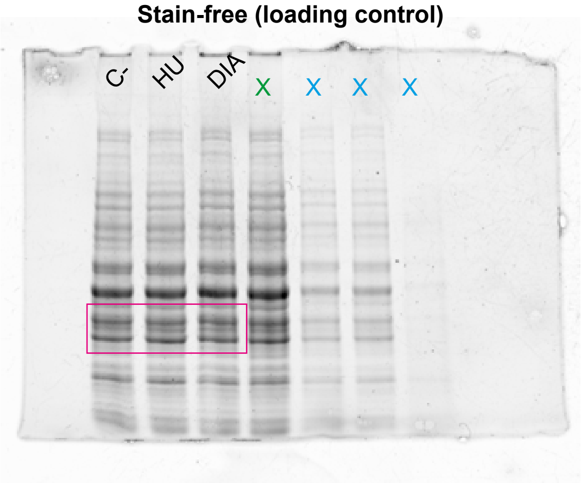

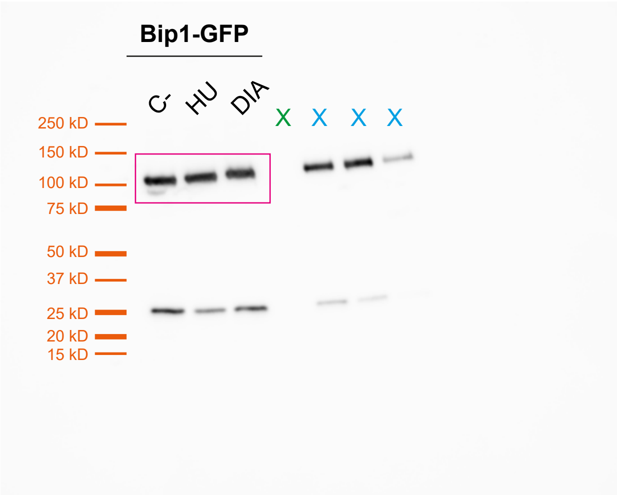

Blots in **Figure 4B** were generated from the blots above (delimitated areas in magenta; the loading control is shown on the left using stain-free detection technology, on the right the blot incubated with anti-GFP is shown).
Lanes with a green X indicate extracts from an untagged wildtype.
Lanes with a blue X correspond to a different experiment not included in the presented figures.

Stain-free (loading control)

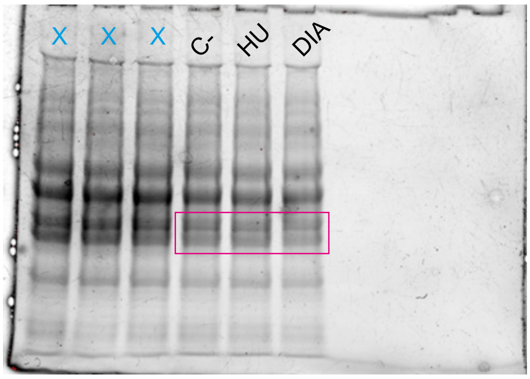

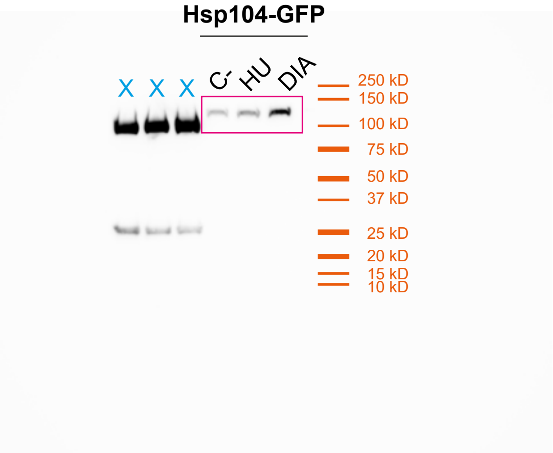

Blots in **Figure 4D** were generated from the blots above (delimitated areas in magenta; the loading control is shown on the left using stain-free detection technology, on the right the blot incubated with anti-GFP is shown).
Lanes with a blue X correspond to a different experiment not included in the presented figures.

Stain-free (loading control)

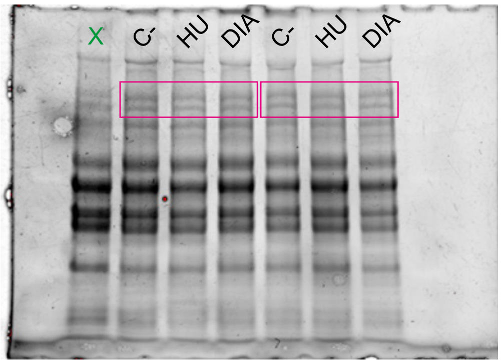

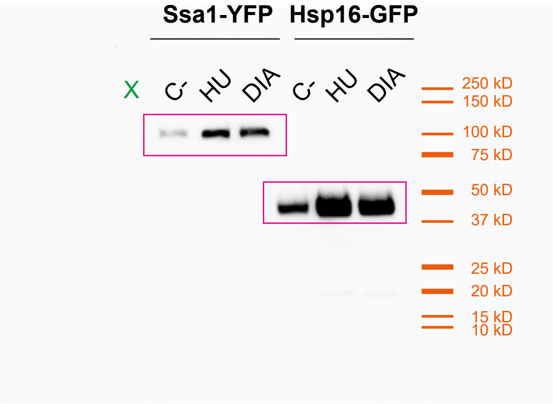

Blots in **Figure 4E-F** were generated from the blots above (delimitated areas in magenta; the loading control is shown on the left using stain-free detection technology, on the right the blot incubated with anti-GFP is shown).
Lanes with a green X indicate extracts from an untagged wildtype.
Lanes with a blue X correspond to a different experiment not included in the presented figures.

Stain-free staining was used as loading control, while protein detection was performed using Supersignal West Femto (Thermo Fisher; Cat. No. 34095). Images were adquired with a Chemidoc XRS+ (Bio-Rad).
